# Bridging the gaps: advancing preconception nutrition in South Asia through evidence, policy, and action

**DOI:** 10.1016/j.lansea.2025.100585

**Published:** 2025-04-24

**Authors:** Faith Miller, Vani Sethi, Avishek Hazra, Danielle Schoenaker, Ranadip Chowdhury, Jane Hirst, Zivai Murira, Naomi M. Saville

**Affiliations:** a Institute for Global Health, University College London, London, UK; b United Nations Children’s Fund (UNICEF) Regional Office for South Asia (ROSA), Kathmandu, Nepal; c Population Council Consulting, New Delhi, India; d School of Human Development and Health, Faculty of Medicine, University of Southampton, Southampton, UK; e MRC Lifecourse Epidemiology Centre, University of Southampton, Southampton, UK; f NIHR Southampton Biomedical Research Centre, University of Southampton and University Hospital Southampton NHS Foundation Trust, Southampton, UK; g Society for Applied Studies, New Delhi, India; h The George Institute for Global Health, School of Public Health, Imperial College London, UK

**Keywords:** Preconception nutrition, Research gaps, Stakeholder interviews, Qualitative analysis, South Asia

## Abstract

This paper summarises the research, policy, and program gaps impeding the advancement of preconception nutrition in South Asia. In line with our evidence reviews, qualitative semi-structured interviews with researchers and programme implementers identified gaps in our understanding of the prevalence and burden of preconception malnutrition due to limited survey and programme data, poor coverage of recommended interventions, and gaps in programme knowledge on effective intervention mechanism. Key barriers identified were the lack of evidence linking preconception care with long-term maternal and child health and nutrition outcomes and how to integrate preconception nutrition interventions into national health systems. We highlight the need for evidence-based, context-specific interventions which utilise effective delivery platforms and engage appropriate actors to reach diverse groups of women and men during the preconception period. We, as part of the South Asia Preconception Nutrition Collective, present actionable recommendations to address these gaps.

**Funding:**

UNICEF Regional Office for South Asia contract number 43384734.

## Introduction

The preconception period, encompassing the time before conception, is increasingly recognised as a critical window for interventions to improve maternal and child health. However, our reviews highlight disparities in the nutritional status among women and girls in South Asia,^1^ the limited evidence on the effect of preconception nutrition interventions on nutritional outcomes at birth or during pregnancy,^2^ and the absence of universal preconception nutrition programs and wide-reaching barriers to implementation into existing health systems.^3^ Furthermore, current research efforts are fragmented, with no consistent definition of the preconception period, inconsistent identification of target populations, and a lack of consensus on the most appropriate delivery platforms, actors, and strategies.^1^^,^^2^ Addressing these gaps requires a comprehensive understanding of stakeholder perspectives, particularly those of researchers and program implementers who are directly involved in the design, development, and rollout of preconception nutrition interventions.

In this paper, we summarise the research, policy and program gaps which exist within the field of preconception nutrition in South Asia by drawing on three sources of information. First, we conducted a qualitative study using semi-structured interviews with researchers and programme implementers in the field to discuss the priorities, challenges and opportunities for advancing preconception nutrition research in the region and identify research gaps. Second, we examined how recommendations arising from our three recent research and policy reviews^1^, ^2^, ^3^ overlapped with stakeholder perceptions. Third, we discussed and validated findings from the reviews and stakeholder interviews with members of the South Asia Preconception Nutrition Collective (SAPNC) during a regional meeting to refine research gaps.

## Methods

### Study design

For this qualitative study, we conducted semi-structured interviews with stakeholders involved in research or program implementation relating to preconception care or nutrition in South Asia. We sought to determine “What are the research gaps on intervention packages in preconception nutrition care in South Asia which are impeding policy and programme action?”

### Stakeholder eligibility and recruitment

Potential stakeholders were identified from the reference lists of our two preconception nutrition reviews^1^^,^^2^ and by leveraging existing professional networks. Stakeholders were eligible for participation if they were i) undertaking or had undertaken research on preconception nutrition ii) implementing or had implemented preconception nutrition interventions in South Asia, or iii) a global expert in preconception nutrition, who could provide insights on preconception research priorities in South Asia. We created a prioritised list of 64 potential stakeholders who we contacted via email and invited to take part in a 1-h interview. Thirty responded, four refused, twenty-six agreed in principle, and twenty-three were available for interview within the timeframe. 19 respondents agreed to be named (provided in Supplementary file S1). Three were programme implementers (one from Bhutan and two from India) and 20 were involved in research in India, Nepal, Bangladesh, Pakistan and Vietnam (8, 6, 3, 3 and 1 respondents respectively). No respondents from Sri Lanka were available and none were identified in Afghanistan and the Maldives. One was a representative of the International Federation of Gynaecology and Obstetrics (FIGO) and one represented both FIGO and the Federation of Obstetric and Gynaecological Societies of India (FOGSI).

### Interview procedures

Twenty-three interviews were conducted online in May–June 2024 via Microsoft Teams or Zoom by either FM or NS. We provided participants with the information sheet and topic guides before the consultation via email, and obtained consent prior to the interview. We conducted interviews in English, recording interactions if participants provided consent. We obtained ethical approval from University College London (ID 27403/001). We developed topic guides, informed by the findings of our recent reviews^1^^,^^2^ and asked respondents to reflect on i) experiences in preconception research and/or programmes, ii) perceptions on the main nutritional challenges in the preconception period, iii) how to define the preconception period, iv) how to reach premarital adolescents, married couples and couples between pregnancies, v) priority research questions, vi) descriptions of interventions that need testing, vii) how to integrate interventions into national health, education and social protection platforms, and viii) policy issues affecting the roll-out of preconception nutrition interventions.

Topic guides are provided in Supplementary file S3.

### Data analysis

We generated transcripts generated automatically by Microsoft Teams or Zoom. FM and NS reviewed, edited and anonymised transcripts. We coded transcripts deductively using the coding framework in Supplementary file S4, updating the framework iteratively if additional themes were identified which did not fit the framework. A collection of quotes from the interviews mapped onto the coding framework is provided in Supplementary file S5. We followed the methods described by Attride-Stirling^4^ to abstract common themes from the data coded into the framework and generate two thematic networks. This involved identifying the two predominant ‘high-level themes’ and grouping the codes into three ‘organising themes’ under each of these. Within each of the organising themes we grouped issues that stakeholders felt require further investigation as ‘basic themes’. We did not make a shortlist of questions or undertake a prioritisation exercise (Supplementary file S4).

### Validation with the South Asia Preconception Nutrition Collective

We validated our analysis by presenting the findings from the 23 stakeholder interviews, together with findings from our evidence reviews^1^, ^2^, ^3^ to preconception researchers and program implementers at the South Asia Preconception Nutrition Collective (SAPNC) meeting, held in Delhi on 21–22 November 2024. A list of participants is provided in Supplementary file S2. Forty in-person representatives came from universities and medical health research centres in India (n = 21), Bhutan (n = 1), Sri Lanka (n = 1), Nepal (n = 1), and UK (n = 3), UNICEF India (n = 11) and UNICEF Regional Office for South Asia (RoSA) (n = 2). Online representatives joined from universities, government and UNICEF in Sri Lanka, Nepal, Pakistan, Bangladesh, Afghanistan, Bhutan, India and UK (n = 22). These people were chosen because they were involved in preconception research or programming in the region. During this meeting, members critically evaluated the key messages and recommendations derived from our analysis. Following a robust discussion, members provided several additional recommendations, which we subsequently incorporated into the analysis.

### Reflexivity

While the researchers conducting the interviews and analysis (FM and NS) are not South Asian, NS has lived and worked in the region for 30 years. The broader research team comprised a mix of South Asian and non-South Asian coauthors. To ensure cultural relevance, stakeholders were predominantly South Asian, with experience in preconception nutrition research in the region. We presented and validated study’s findings in a workshop with South Asian experts in preconception care. Most coauthors are predominantly researchers by training, which may have limited our perspective on the practical barriers faced by frontline implementers.

## Results

**Fig. 1:**
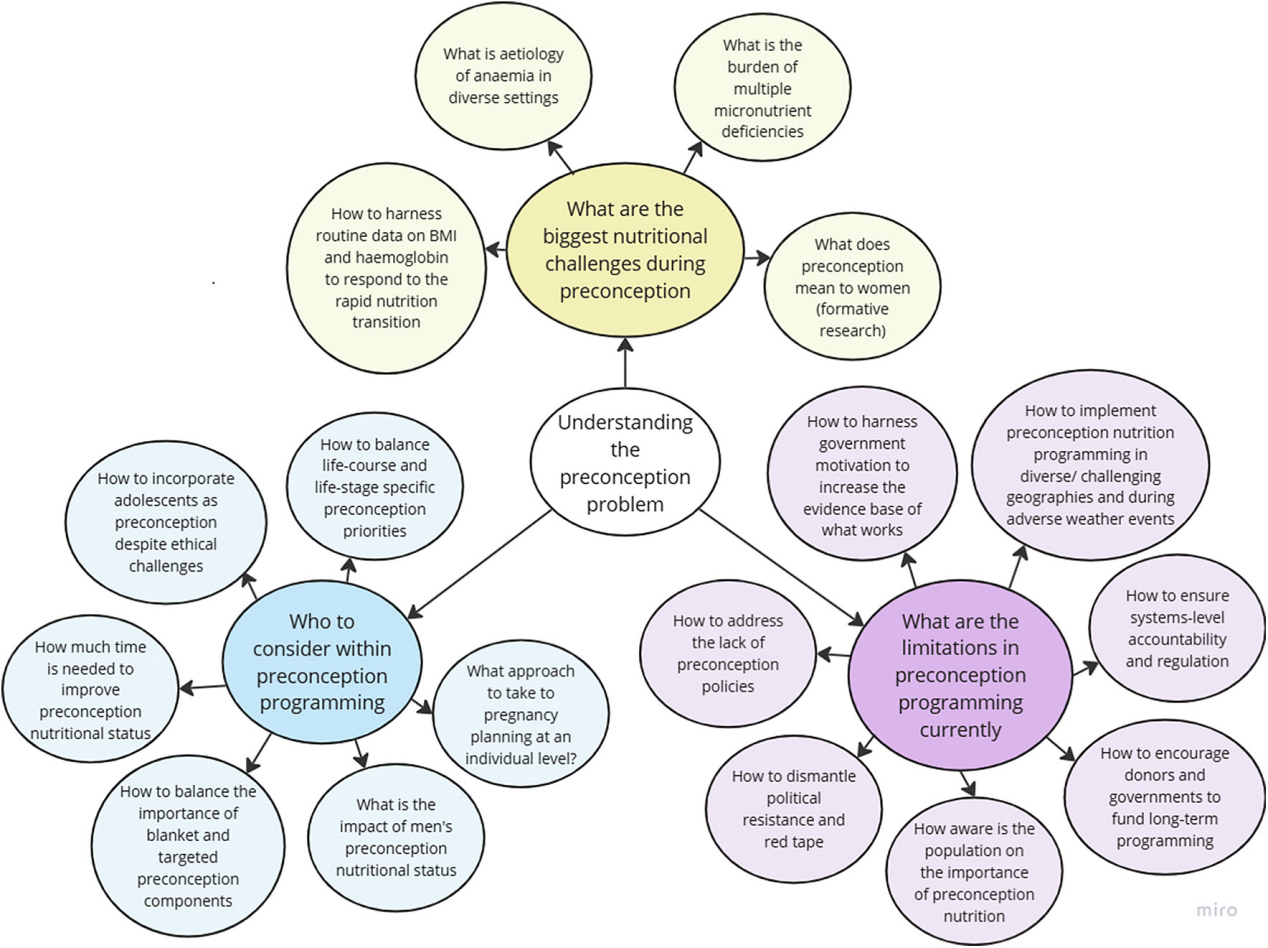
Thematic network of basic and organising themes within the high−level theme A of “Understanding the preconception problem”.

**Fig. 2:**
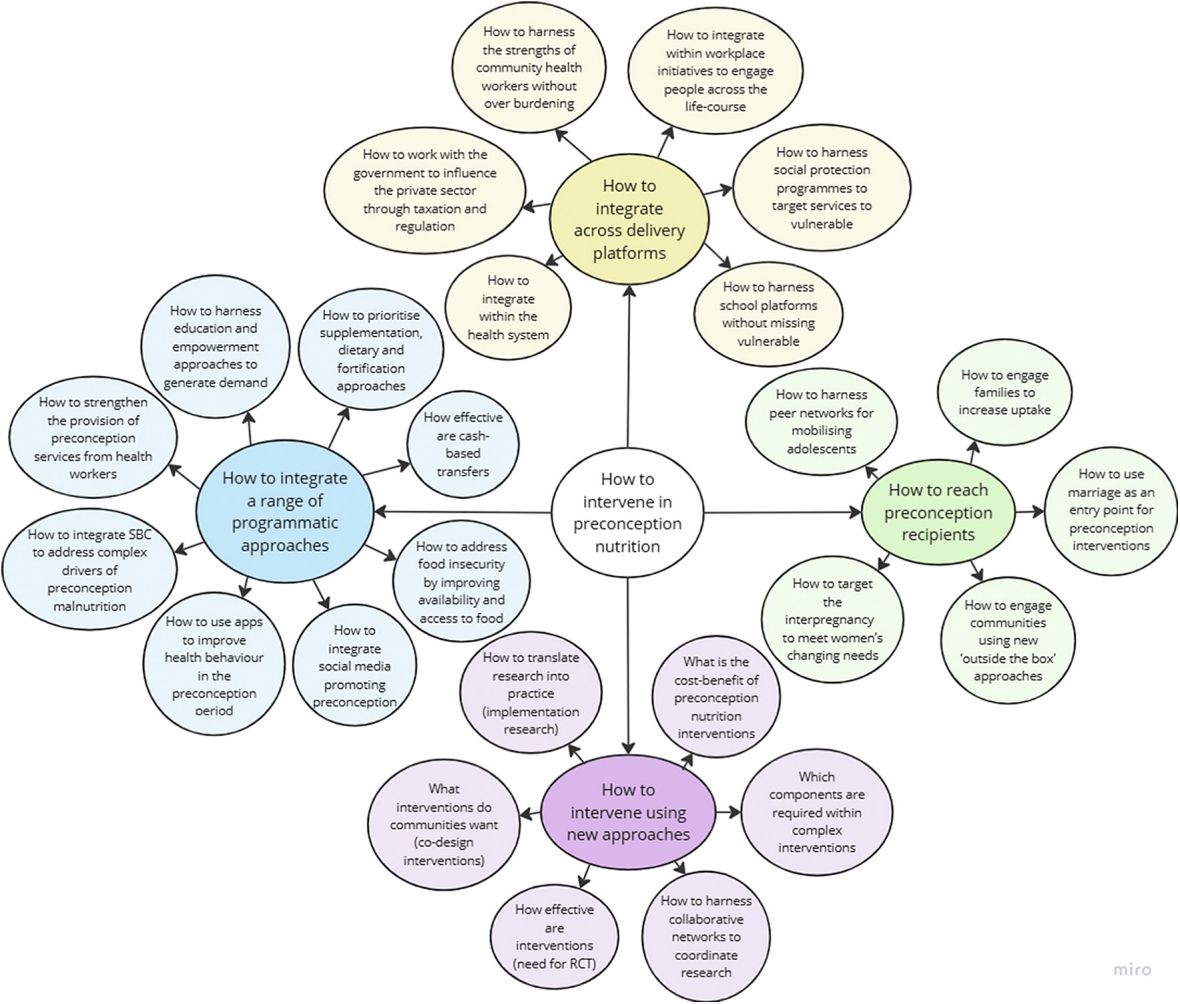
Thematic network of basic and organising themes within the high−level theme B of ‘How to intervene in preconception nutrition’.

We present findings grouped by the organising and basic themes from stakeholder interviews as summarised in Figs. 1 and 2. Further details are provided in Supplementary files S4 and S5. The most commonly reported research priorities were measuring nutritional status, exploring micronutrient or food supplements, dietary interventions, how to target the most vulnerable, what preconception means to women, integrating social and behaviour change (SBC), and how to cope with the changing needs as the nutrition transition unfolds. We also discuss interview findings alongside research gaps from our research and policy reviews.^1^, ^2^, ^3^ Global and organising themes are presented as headings below, and basic themes (in inverted commas) are described under each of the organising themes. Supportive evidence (verbatim quotes) for each of the themes are provided in Tables 1 and 2 and referred to in the text.

**Table 1:** Quotes from researcher stakeholders under the high−level theme A: “Understanding the preconception problem”.

Organising theme	Basic theme	Quotes from stakeholders	
1. Who to consider within preconception programming	1.1 How to balance life course and life-stage specific preconception priorities	*“Preconception goes all the way back to fetal life, right?” [P17].*	
	1.2 How to incorporate adolescents as preconception despite ethical challenges	*“If I was a young girl, I wouldn’t want people to come to me and treat me like a walking, talking uterus. Which is what preconception implies.” … “I would take conception out of it”,* and when referring to their own work: *“we didn’t use preconception care as a term in that even once … because I felt very strongly that, you know, we should not really couch all … young girls as mothers in waiting.” [P21]*	*[adolescence] that’s a very important stage, which actually sets a trajectory for their own health. So, I think rather than just see them as a preconceptional target audience, you know, I think they’re a very important group because that’s when we can really change trajectories then.” [P23]*
	1.3 What is the impact of men’s preconception nutritional status	*“One of the reasons which is affecting sperm quality is zinc deficiency … we do not know any idea about the nutrient status of men in our country or adolescent boys … So, there can be like more research in that area” [P01]*	
	1.4 How much time is needed to improve preconception nutritional status	*“Things like overweight and obesity, you can’t shift easily, so you need more time … Some people can stop smoking or change their diet very quickly, but for many people they can’t. And also, you can’t shed lots of weight very quickly for sure” [P12]*	
	1.5 What approach to take to pregnancy planning at an individual level?	*And I guess the depth of material, so you could start telling people about concepts when they’re younger, but then get it more, once it becomes more real to you. And we’re like, it’s in your future, then you get a bit into more depth, but at least you’ve sort of primed someone. I guess they’re a bit aware of the concept from earlier on, and I guess when you’re younger you probably want a bit more about maintaining good health in your own health and well-being and how to have appropriate nutritional intake. Versus when you’re a bit older it’s more about planning to have a baby and what should you do in advance of becoming pregnant? What are? What are things that you can do? [P22]*	
	1.6 How to balance the importance of blanket and targeted preconception components	*“When you’re younger you probably want a bit more about maintaining good health in your own health and well-being and how to have appropriate nutritional intake. Versus when you’re a bit older it’s more about planning to have a baby and what should you do in advance of becoming pregnant?” [P22]*	*“In my opinion, you wanna tailor your supplement to what’s actually there. If iron deficiency is not actually an issue, then you probably don’t wanna give people iron, cause that can have really big side effects and that can sort of undermine your entire supplementation regimen.” [P22]*
2. What are the biggest nutritional challenges during preconception	2.1 What does preconception mean to women (formative research)	*“Get [the local community’s] perspectives on the study, what their needs and priorities were” [P23]*	
	2.2 What is the burden of multiple micronutrient deficiencies	*“The peculiar problem of the double burden that we have, you know, both underweight and overweight and then micronutrient deficiencies, which are common in both groups.”* [P23]	
	2.3 What is aetiology of anaemia in diverse settings	“*When we say anaemia, we just think it’s iron deficiency …. what we found from our work is that … just around 50% of the women have iron deficiency*” [P19]	“*Women with haemoglobinopathies present with low haemoglobin but high circulating iron, and they are at risk of iron overload and then presenting with cardiac problems and heart failure*.” [P19]
	2.4 How to harness routine data on BMI and haemoglobin to respond to the rapid nutrition transition (over- and underweight together)	“*It’s a rapid transition that’s happening, which is going to have major impacts upon preconception nutrition year by year. I mean we just found … that the proportions of women and of small children consuming unhealthy deep fried foods and sugar sweetened foods over a 2 year period just leapt up.”* [P10].	
3. What are the limitations in preconception programming currently	3.1 How aware is the population on the importance of preconception nutrition	“*I don’t think the barriers are that problematic. Actually, I think you just need to make people aware of it because obviously it’s not something that’s been on people’s radar very much.”* [P12]	
	3.2 How to implement preconception nutrition programming in diverse/challenging geographies and during adverse weather events	“*We are very mountainous, difficult geography country with a very difficult and scattered settlement, and sometimes we have monsoon, we have flash floods and then roads are cut off and then supply chain is disrupted. So, we do have these kinds of challenges … which is one reason why people eat, you know these non-perishable staples.”* [P18]	“*You know, there’s still that seasonality, but now the effects are stronger and more intense. The rhythm is still there, but the amplitude of the exposures I think is changing and how to deal with those exposures and understand what they mean for different outcomes is really complex*.” [P14]
	3.3 How to ensure systems-level accountability and regulation	“*There is no accountability in the system. Just you need to distribute ….Once they come, what has happened, whether the mother had received, whether you see [her] taking. So, there is no real accountability on the part of the system.”* [P04]	
	3.4 How to harness government motivation to increase the evidence base of what works	“… *for policymakers, they want to know… What is the evidence why you are saying that this is important?”* [P13]	“*There is a huge gap of knowledge and awareness among the policymakers and the government when it comes to preconception nutrition. So … spreading the science behind why it is really required*.” [P06]
		“*Feeding programs are showing modest effects, but honestly they’re not very impressive. I mean, they’re not solving the problem”* [P08]	
	3.5 How to address the lack of preconception policies	“*The main policy issue is that there is no preconception system right now as a system level. So that remains the prime challenge.”* [P03]	“*There was no clear guideline which was given by the government … when it came to preconception nutrition*” [P06].
	3.6 How to encourage donors and governments to fund long-term programming	“*We all live in three to five year intervals, right? When it comes to grant funding, so you can’t do a long term study with one grant.” … “donors are resistant to thinking about this, and … we need to get them on board with this process*.” [P08]	“*You know, electoral cycles are short …, the truth is, governments don’t think a long time ahead*” [P09]”
	3.7 How to dismantle political resistance and red tape	“*The other very important issue is, you know, delays that happens in the government system in implementing the trial because of the red tapeism, you know there are many blocks. If a particular decision has been taken and if it has to be implemented, a decision has been taken to procure something then you have multiple obstacles which delay. And by the time you procure … the intervention that you want we must have wasted the whole time of the intervention or the study duration. So that is, I think these are very important. From the point of rolling out*.” [P04]

**Table 2:** Quotes from researcher stakeholders under the high−level theme B: “How to intervene in preconception nutrition”.

Organising theme	Basic theme	*Quotes from research stakeholders*	
4. How to integrate a range of programmatic approaches	4.1 How to prioritise supplementation, dietary and fortification approaches	*“I have no confidence that dietary interventions are going to make any difference … You wanna make an efficacy statement first [to] prove that the principle actually works before you start thinking about programmatic approaches to improving nutritional status in women” [P08]*	*“Yeah, I mean we also toyed with the idea of giving these lipid based supplements in a calorie rich food and then we decided not to go down that route … because it becomes a product. It’s not, you know, we didn’t think it was food … I don’t have all the answers, but I think [dietary approaches are] what I would expect to see in the longer term rather than just give everyone a pill.” [P23]*
		*“… multi-pronged approach [of] direct and indirect elements as well. So, supplementation being something that’s direct, but then food fortification would probably be something that sort of gives everybody that little bit of a bump in their intake.” [P22]*	
	4.2 How to harness education and empowerment approaches to generate demand	*“Storytelling [in] the local language which they really understand, what they relate to, that should be done in order to spread awareness and education on [preconception nutrition] and maybe even a pictorial sort of a thing can be taken into consideration, and they can [do] an enactment in front of everyone in order to promote that kind of education or awareness about preconception nutrition.” [P06]*	
	4.3 How to address food insecurity by improving availability and access to food	*“It can be challenging for sure because you don’t necessarily have access to foods. So, if you’re teaching someone about what they should be eating, but they can actually put it into practice that’s difficult.” [P22]”*	*“Foods available that are healthy and nutritious and have micronutrients and have protein, but poverty is such a huge challenge in that setting, that actually affording them is a different situation altogether.” [P22]*
	4.4 How effective are cash-based transfers	*“I think it puts more income into a household, and you would hope that would sort of translate into improved diets of the girls and perhaps boys preconceptionally. But it’s sort of, it seems like a more diffuse pathway.” [P11]*	*“Until we have some evidence that in a controlled setting it really works, all that stuff has the potential to kind of just get washed away and you see no impact, right? Did you not see any impact? You have no idea what the story is, right? You don’t know why there wasn’t an impact.” [P08]*
	4.5 How to integrate social and behaviour change (SBC) to address complex drivers of preconception malnutrition	*“The cultural belief and practice is also something we need to consider because a lot of regions is some belief [of] what [to] consume, what not [to] consume … Plus if they add the daughter in law … there’s some kind of accessibility and disparity [within the] household? Because sometimes the daughter cannot eat all the good food but the mother in law and the husband need to eat first” [P20]*	*“So many of these misconceptions … need to be, you know, taken into account for a robust implementation … To put the policies may be easier than implementing it as … more barriers will stand in the way of the nutrition related [research] because it is so behaviour based” [P05]*
		*“Communities will do what communities will do. And they will do things that they perceive to be of value and for reasons that are very different from what you want them to do. And I think one big challenge there was, I think, placing everything in the basket of birth outcomes and others for young girls, you know, makes the whole thing very unattractive in the context of their own aspirations of life and what they want.” [P21]*	
	4.6 How to integrate social media promoting preconception	*“The electronic media and all the social media, you know, is very powerful. Every household at least have the mobile phone and the TV” [P15]*	*“There is also a lot of drive and because of all the social media and all this and people have access to all sort of information, some incorrect.” [P18]*
	4.7 How to strengthen the provision of preconception services from health workers	*“We have to build the capacity for that and use the electronic space or digital space very cleverly to position some of these awareness initiatives and to make them realize that you must ask this question to your doctor.” [P05]*	*“Then the person needs to be able to say something. Then the care provider needs to be confident and equipped to say something sensible” [P12]*
	4.8 How to use apps to improve health behaviour during the preconception period	*“In this era of the digital space, [healthcare providers] can always say ‘OK, go and seek out from this app, you’ll get your recipes, you’ll get your know how of how many calories, what you’re eating’. The person, you know has the opportunity to go in depth … the demand is easier to generate because people do want to take care every day” [P05]*	
5. How to integrate across delivery platforms	5.1 How to harness the strengths of community health workers without over burdening	*“The adolescents out of school will be in the community. The community. Soon to be married adolescents, just married, in between pregnancy. They’re all in the community.” [P04]*	*“Ownership plays a very important role. So, ensuring that ownership is also there at the ground level amongst the healthcare workers who are trying to promote these things should be there.” [P06]*
		*“We would probably be using the same community health workers to deliver [a preconceptional program], but they’re already quite busy. You know, they have a lot on their plate, so if we have to add preconception nutrition to them, then do we have additional, do we have additional resources to recruit more staff?” [P23]*	
	5.2 How to harness school platforms without missing vulnerable out of school adolescents	*“I think definitely school-based platform is some place that you can reach them more effectively with mass amount of people that way “[P20]*	*“The vast majority of these kids are coming in without any breakfast when they show up at school. So, you’ve got to do something kind of early in the morning to make sure [that] actually helps them stay awake and pay attention during school” [P08]*
		*“50% women are married before 16.3 years of age … [and] 75% of women who got married, they stopped their education. So that’s why, like targeting through school based platform or education based platforms [is] a bit problematic for our case” [P01]*	
	5.3 [Evidence for organising theme]	*“If you do it within a life course framework [with] programmatic entry points which might be in different services, you can have those conversations [while] avoiding all this sort of guilt tripping” [P12]*	
	5.4 How to integrate within the health system (particularly family planning) to ensure continuity	*“It is easy to put into, integrate into a national health system. Because you have already existing the infrastructure and the human resources. Yeah, so it would be much easier to integrate that into a national existing national health programs, or the system.” [P04]*	*“For me it’s just very simple. Two sides of the same coin: trying to help people avoid the pregnancies they don’t want and have the pregnancies they do want in the best kind of health, best shape possible” [P12]*
		*“Somebody comes with heavy menstrual bleeding; they only fix the heavy menstrual bleeding. It is leading to anaemia. They’re not thinking about anaemia and nutritional counselling, for example” [P05]*	*“Few adolescents, if any, go to the health system. They are generally very reluctant in terms of going to, you know, facilities with obstetricians and gynaecologists. No young girl wants to be seen outside in a gynaecologist’s office, and neither do their parents want them to be there.” [P21]*
	5.5 How to harness social protection programmes to target services to vulnerable	*“So, I do think that there is a need for a range of strategies, poverty alleviation and education being a dominant one, but then also including things that improv[e] dietary diversification through a range of strategies like for example cash transfers, linking them to poor families” [P21]*	
	5.6 How to work with the government to influence the private sector through taxation and regulation	*“Taxation type of thing might be a way into policy, because obviously it raises money. So, then you can begin to talk about how the funding that was raised by such and such programs could be deployed to train community health workers or whatever it is.” [P09]*	*“So that you’re not asking people to really change their behaviour. Makes it easier, asking governments and companies to change their behaviour,” [P11]*
	5.7 How to integrate within workplace initiatives to engage people across the life course	*“Girls’ economic empowerment is an important area. And so there could be such platforms where there, you know, the skill building is being done. Or yeah, there could be those types of platforms that we could think about as workplace opportunities.” [P14]*	
6. How to reach preconception recipients	6.1 How to target the interpregnancy period to meet women’s changing needs	“*If you had enough time and money, I would want to look at … the interpregnancy interval as well because you know, we know their pregnancy depletes women’s nutrients status and … we could supplement them through to the next pregnancy*” [P11]	
	6.2 How to engage families to increase uptake	“*For any kind of … program, it is very important to involve the husband and the other family members, because sometimes in this part of the world they are the key decision makers of the family.”* [P13]	“*Culturally it’s not very common for the men or the husbands to get involved in the care of their wives during pregnancy or in the care of the children when they are very young. You know, that’s mostly done by the mother and the grandmother, but we actually discovered that many husbands wanted to get involved. And it was just a question of them not being able to know how to get involved*” [P23]
	6.3 How to use marriage as an entry point for preconception interventions for newly weds	“*The newlywed couples … they register immediately and they come for screening. I’ve seen how Sri Lanka does it. So, after marriage, they just come and then … I think they do get screening for anaemia, BMI and all of that ….”* [P19]	“*In Nepal with the FCHV program, those guys, they know their people, they know who’s getting married and so on.”* [P11]
		“*It’s challenging because of these new rules around age at marriage and people wanting to hide that they’re marrying their girls early … I feel that there needs to be a marriage registration system so that communities are able to identify newlyweds and without the fear of retribution and legal action and all of that. Because I feel like that is actually a bit of a barrier to the most vulnerable getting services.”* [P10]	
	6.4 How to harness peer networks for mobilising adolescents	“*Having workshops along with the peer group … I think will help in educating them better because if you are educating the peers, you’re also educating the all the other peers around them, when it comes to that age group, you know, so you can sort of motivate them and encourage them to have a behavioural change when it comes to these things. So that would be the adolescent group part*” [P06]	
	6.5 How to engage communities using new ‘outside the box’ approaches	*“I think it we need to think out of the box. We cannot be stuck in the same process and the same cycle of thinking and just the same pattern, and I’m sure a lot of people will have loads of these kind of ideas which you have to I think try and probe and get out, however weird it might seem, this is where you get all the sparks*.” [P19]	
7. How to intervene using new approaches	7.1 How to harness collaborative networks to coordinate research	*“We have identified the … champions, and the people are really willing to do this work, [we] just need to come in contact with somebody who is able to then give that funding for this network to do the work. It has to be a South Asia wide collaboration, not one individual.” [P19]*	
	7.2 How to translate research into practice (implementation research)	*“The research I think now is implementation research. I think we know what needs to be done. The question is how can we do it and what works?” [P09]*	*“A state can’t implement the full [research intervention package] in a public health program without … doing an implementation research, because there are many components.” [P13]*
	7.3 What interventions do communities want (co-designing interventions)	*“I think is it’s very important to work with [adolescents] to actually get them to co-develop the interventions with us. And that’s something again we have discovered through some of the formative work … by working with them and helping the women arrive at their own solutions and what they think will work in their setting and in their specific circumstances.” [P23]*	
	7.4 Which components are required within complex interventions	*“We were seeing women who had multiple challenges so focusing on just nutrition alone probably wasn’t the solution [and] therefore we decided to have a multifaceted intervention which focused on nutrition, maternal, mental health, pollution, hygiene, and parenting skills and child development, all underpinned by behaviour change” [P23]*	*“When designing a complex intervention, you need to kind of come up with a theory of change of how women end up having these preconceptual nutrition problems in the first place, and then would be what are the various pathways by which you might influence them” [P10]*
		*“We delivered a package, and that package worked. Now, which component of the package worked? That is very interesting to understand.” [P13]*	
	7.5 What is the cost-benefit of preconception nutrition interventions	*“What is the cost for that? Because everything has cost. Again, I’m saying so two things. What is the evidence and what is the way that evidence is cost effective or not?” [P13]*	*“The first question is ‘what does it cost?’ And it’s too expensive. Immediately … I think political will in general for women’s nutrition–women and girls’ nutrition is lacking! I just feel like it’s not, it’s not an affordability issue in many contexts. Some of [the governments] in South Asia should be able to afford to do better with women’s health and nutrition, and it’s just of the lack of, you know, commitment and political will.” [P14]*
	7.6 How effective are interventions (need for randomised trials)	*“[Trials are] very likely to have to be cluster randomized … for acceptability purposes.” [P08]*	

### Understanding the preconception problem

#### Who to consider within preconception programming

As a collective, we define women in the preconception period as non-pregnant women and girls aged 15–49 years who have not undergone sterilisation, as per the WHO definition,^5^ regardless of marital status, parity, or fertility intention. However, when we reviewed the published literature on the burden of preconception malnutrition, the age range used varied widely.^1^ This lack of consensus was reflected in the perspectives of the 23 stakeholders we interviewed, who had different opinions on how to define the preconception period (Fig. 1). We organised an expert group consultation in 2023 to discuss the definition of preconception and who to target as preconception women. The group suggested that the preconception period and population can be defined from theoretical and programmatic perspectives. From a theoretical perspective, it is important to consider a broader continuum of care for preconception nutrition, starting in adolescence, rather than limiting the focus to just 3 months to 1 year before conception. This approach views both men and non-pregnant women aged 15–49 years as part of the preconception population, recognising that comprehensive care across this span supports better nutritional and health outcomes before conception. From the programmatic and practical viewpoint, experts suggested focusing on specific groups: i) pre-pregnancy individuals, including newlywed couples and nulliparous women, and ii) women or couples in the interpregnancy period; with an age group of 15–35 years or 15–49 years. This multipronged approach covers both adolescents and women likely to conceive, creating opportunities to improve preconception, maternal, and infant nutrition most effectively.

In line with the expert group opinions, interview participants highlighted tensions within the research community around ‘How to balance life course and life-stage specific preconception priorities?’ [1.1, P17] and ‘What approach to take to pregnancy planning at an individual level?’ [1.5, P22]. A life-stage specific approach targets individuals when they start planning to have a baby, enabling services to be tailored depending on the nutritional needs of the individual, in line with the individual perspective recommended by the Lancet series on preconception health.^6^ However, this approach prevents interventions from reaching people having unplanned pregnancies and provides a limited window to intervene for planned pregnancies. Alternatively, a life course approach aims to improve the health of everyone who has any chance of becoming a biological parent later in life, to improve their health as individuals as well as the health of future generations, in line with the public-health perspective recommended by the Lancet series on preconception health.^6^ Early life interventions kick-start positive health trajectories and behaviours. However, this wide window for intervention makes it difficult to target programs to maximise impact and poses ethical concerns as not all reproductive age people are able to or plan to become parents. Some stakeholders suggested removing any mention of the word ‘conception’ from preconception programming to avoid considering all young girls as future mothers [1.2, P21]. This is further complicated when considering ‘How to incorporate adolescents as preconception’, as classifying adolescents as preconceptual places value on their role as producers of future generations rather than individuals with their own right to health [1.2, P21, P23].

Determining ‘how much time is needed to improve preconception nutritional status’ before conception is an ongoing challenge and varies by outcome. For example, losing weight takes longer than addressing specific micronutrient deficiencies [1.4, P12]. Our review of preconception interventions also highlights that research is needed to determine how to tailor program duration, and that this must be based on its intensity.^2^ When programs have a longer duration and intensity, community engagement and health system strengthening is also required.^7^

Another research gap is ‘how to balance the importance of blanket and targeted preconception components’. Blanket interventions target all individuals within a certain group but raise issues if side effects undermine adherence, as is reported with blanket IFA supplementation among adolescents [1.6, P22]. Furthermore, the evidence for blanket and targeted approaches to preconception nutrition depends on the nutritional needs of the population.^2^ For example, two preconception food supplementation trials which took place in India (providing lipid nutrient supplements and an enhanced micronutrient content locally prepared snack), found that supplementation must begin at least 90 days before conception to improve women’s nutritional status in this context.^8^^,^^9^

Although most interview participants primarily referred to the health of women during preconception, the question of ‘what is the impact of men’s preconception nutritional status?’ was highlighted as demanding further research because data are lacking on preconception men’s nutritional status despite emerging evidence that men’s nutritional status can affect sperm quality [1.3, P01].

#### What are the biggest nutritional challenges during preconception

A key research gap reported by interview participants is the need for formative research on ‘what preconception means to women’ to deepen our understanding of the culturally driven social and gender norms relating to nutrition [2.1, P23]. This is in keeping with recommendations from our review of preconception nutrition interventions, which highlighted the need for community consultations to inform pathways to implementation.^2^

Despite trials and reviews of multiple micronutrient supplementation (MMS), research gaps remain around ‘the burden of multiple micronutrient deficiencies’ [2.2, P23]. National data on micronutrient deficiencies is not available for most of South Asian countries and there is large heterogeneity in methods used to determine anaemia and micronutrient deficiencies regionally.^1^ Our review on the burden of preconception malnutrition also highlighted the persistently high burden of anaemia, demonstrating the need for research exploring ‘what is the aetiology of anaemia in diverse settings’ to inform the design of interventions [2.3, P19].^1^ As also highlighted in this review, iron deficiency only accounts for part of the anaemia burden and therefore iron therapy is not effective to resolve anaemia for all women, such as those with haemoglobinopathies [2.3, P19]. A central finding of stakeholder interviews and our review was that large-scale longitudinal surveys among non-pregnant women of reproductive age are needed to track how nutritional status changes over time throughout different life stages, supporting regional evidence on the lack of harmonised, routine data collection.^1^ Stakeholders stressed that we need to understand ‘how to harness routine data on BMI and haemoglobin to respond to the rapid nutrition transition (over- and underweight together)’. As intakes of unhealthy foods are dramatically increasing, studies on diets and drivers of food choice are particularly important [2.4, P10].

#### What are the limitations in preconception programming currently

Interview participants shared challenges in implementing and researching preconception programmes and frustration around the insufficiency of evidence on what works. In many settings we do not know ‘how aware the population [is] on the importance of preconception nutrition’. However, in settings where services are available, people are generally open to receiving preconception care once they are aware of it [3.1, P12]. Our review of policy and program coverage highlighted that most countries, except Sri Lanka, do not have universal programmes for health and nutrition screening and essential micronutrients specifically during the preconception period, despite available policies.^3^ Interventions to treat and care for at-risk women are missing in most countries. ‘How to address the lack of preconception policies’ was commonly as a challenge [3.5, P03].

To address the lack of policies, an understanding of ‘How to harness government motivation to increase the evidence base of what works’ is needed, given the lack of knowledge and awareness of preconception nutrition among policy makers [3.4, P13, P08, P06]. Where policies are in place, there is a challenge in ‘how to ensure systems-level accountability and regulation’ [3.3, P04], because of a lack of accountability among government health workers. Our review of policy and program coverage also highlighted that several of the countries in the region face moderate to severe system-level bottlenecks in program implementation, especially in budget and financing, data and information, workforce, supplies, and service delivery.^3^ Implementors need to resolve ‘how to dismantle political resistance and red tape’ as this holds back intervention implementation [3.7, P04]. Furthermore, since many parts of South Asia have challenging terrain, monsoonal climate, and frequent extreme weather events, we need research to understand ‘how to implement preconception nutrition programming in diverse/challenging geographies and during adverse weather events’, especially in the context of climate change [3.2, P18].

Meanwhile, ‘How to encourage donors and governments to fund long-term programming’ is an area that warrants investigation and renewed efforts [3.6, P08, P09]. Political agendas are not conducive to long-term programming of preconception health regarding the impacts on the next generation. This was also mirrored in the regional review, which identified only three studies from South Asia exploring associations between preconception nutrition and pregnancy or birth outcomes.^1^ The regional review of preconception interventions highlighted how researchers of the a preconception food supplementation trial using locally prepared micronutrient-rich snacks recommend longer follow-up of participants to explore longer-term functional health effects.^2^^,^^9^ This requires long-term investment and commitment from governments and research funding agencies.

### How to intervene in preconception nutrition

#### How to integrate a range of programmatic approaches

Our policy and program bottleneck analysis revealed that social norms hinder the implementation of interventions such as psychosocial screening and family planning to delay first pregnancy.^3^ While SBC communication approaches have been widely used for improving awareness and practices around maternal, newborn and child health, ‘How to integrate SBC to address complex drivers of preconception malnutrition’ was stakeholders’ most common question around interventions (Fig. 2). To design effective SBC, research must first determine the complex and diverse drivers of malnutrition in order to investigate how to improve consumption of a healthy diet, encourage physical activity, address food taboos, and challenge harmful gender norms at the community and household level [4.5, P20, P05, P21].

A central strategy for the prevention of anaemia among women in the preconception period regionally is the roll-out of IFA supplementation among adolescent girls. However, our review of preconception interventions found inconsistencies in the association between adherence to IFA supplementation and anaemia prevalence.^2^ This may be explained by the range of indicators used to represent adherence, including receipt^10^^,^^11^ or consumption of IFA tablets during different time periods.^12^, ^13^, ^14^, ^15^, ^16^ There were conflicting opinions among stakeholders on ‘how to prioritise supplementation, dietary and fortification approaches’. Some felt that trials of proof of concept were needed for micronutrient supplements and fortified foods [4.1, P08]. Particular strategies which require further evidence, include the effect of Vitamin A and MMS starting in adolescence,^17^ the effect of B12 supplementation among populations consuming largely vegetarian diets,^18^ the cost-effectiveness and operational feasibility of periconceptual supplementation on anaemia in pregnancy and risk of neural tube defects,^19^ and the association between iodine supplementation before pregnancy and iodine-creatinine ratio during pregnancy and offspring neurodevelopment.^2^^,^^20^

Others were hesitant to recommend research providing supplements and thought dietary approaches were the most sustainable way forward [4.1, P23]. One researcher suggested combining food supplements with large scale food fortification [4.1, P22]. We identified ‘How to address food insecurity by improving availability and access to food’ as a prerequisite for sustainable improvements in nutritional intake, as affordability is a huge concern among vulnerable populations in South Asia [4.3, P22]. Studies also need to investigate ‘how effective are cash-based transfers’ in improving preconception nutrition [4.4, P11], as this is currently lacking evidence.

‘How to harness education and empowerment approaches to generate demand’ is another key question [4.2, P06]. Interventions need to inform women and their families about the importance of improving nutrition during the preconception period. Research exploring the effectiveness of community-based approaches such as storytelling and street theatre are needed [4.2, P06]. Exploring ‘how to integrate social media promoting preconception health’ was also identified as an important component, which needs to be integrated with improved quality of care to meet the demand generated. However, misinformation via social media can be problematic [4.6, P18]. Apps could also be designed to address preconception nutrition to enable people to improve their own health [4.6, P15].

Research to explore ‘how to strengthen the provision of preconception services by health workers’ is needed to enable them to provide good quality, appropriate preconception care [4.7, P05, P12]. This should be supplemented with studies on ‘how to use apps to improve health behaviour during preconception’, enabling people to seek their own health-related information and act upon it [4.8, P05].

#### How to integrate across delivery platforms

To address the complex drivers of malnutrition and sensitivities around discussing the preconception period, integration of interventions across multiple platforms and across the life course is key to provide multiple entry points into preconception nutrition [5.3, P12]. Whilst exploring pathways for improved preconception nutrition, we identified various platforms used in South Asian settings such as field camps, community outreach sessions, and health centres.^2^ These provide health and nutrition screening days, provision of IFA and deworming, and counselling of newlywed couples. Community meetings and home visits by frontline health workers have also been tested for provision of counselling support.

Investigating ‘how to harness school platforms without missing vulnerable out of school adolescents’ was a key question raised in the interviews. Schools provide a useful platform for delivering nutrition education to adolescents as well as delivering initiatives such as school-meals and supplementation programmes [5.2, P20]. However, the most vulnerable adolescents, particularly early married girls, are not in school [5.2, P01]. This was echoed by our review of preconception interventions, which summarised recommendations for school-based interventions including adding preconception care into curricula of schools and promoting consumption of green leafy vegetables through school kitchen gardening, while also delivering education via various platforms, such as worksites^21^ and mobile phone platforms^22^ to ensure equitable coverage.^2^

To reach all women and girls in the preconception period, studies need to encompass ‘how to harness the strengths of community health workers without over burdening’ [5.1, P04], as ensuring community ownership is central to programme success [5.1, P06]. To deal with community health worker burden, additional health workers may need to be recruited [5.1, P23].

‘How to integrate preconception care within the health system (particularly family planning) to ensure continuity of care’ [5.4, P04, P05, P12] was considered another priority. However, across South Asia, barriers to reproductive care-seeking among adolescents persist, highlighting the importance of developing adolescent-friendly services [5.4, P21]. For those in paid employment, we identified the question of ‘how to integrate within workplace initiatives to engage people across the life course’ as an important research and programme gap [5.7, P14].

Investigations of ‘how to harness social protection programmes to target services to the vulnerable’ may address concerns around affordability and access to nutritious foods [5.5, P21]. Stakeholders expressed concerns around the role of food companies in the nutritional transition as consumption of ultra-processed ‘junk’ food increases, which has contributed to increasing levels of overweight and obesity across South Asia. Studies need to address ‘how to work with the government to influence the private sector through taxation and regulation’. Taxation of unhealthy foods was highlighted as a strategy to encourage the promotion of healthier foods [5.6, P09], which may be easier to implement than community-based SBC [5.6, P11].

#### How to reach the preconception population

Stakeholders provided constructive suggestions on how to reach preconception programme recipients at different stages across the life course. Because most pregnancies occur following marriage in South Asia, stakeholders identified ‘how to use marriage as an entry point for preconception interventions for newlyweds’ as a key research area. Marriage registration is a potential trigger for couples to access services [6.3, P19]. In Sri Lanka, this acts as a bridge to link newlywed couples with the health system, where they receive counselling on preconception care. Community health workers could identify newly married couples and deliver preconception counselling [6.3, P11]. A complex preconception and early childhood nutrition intervention that leveraged existing government systems in Maharashtra, India, successfully applied this approach, which has since been scaled-up statewide as the Vatsalya programme.^23^ However, punitive laws to prevent early marriage may pose a problem as families may not declare marriages out of fear of fines [6.3, P10]. Furthermore, low rates of marriage registration are also a hindrance.

‘How to harness peer networks for mobilising adolescents’ both in and out of wedlock remains a priority question, especially because adolescents tend to be most comfortable interacting with their peers, especially around potentially sensitive topics [6.4, P06]. ‘How to target the interpregnancy period to meet women’s changing needs’ is another priority due to women’s engagement with health services during and following pregnancy [6.1, P11]. Interpregnancy efforts should include meeting the nutritional demands of breastfeeding while potentially being in the preconception period with respect to a subsequent pregnancy [6.1, P11].

‘How to engage families to increase uptake’ of preconception nutrition interventions is important regardless of the life stage of the participants, especially as husbands and parents-in-law tend to hold the decision-making power in South Asia [6.2, P13]. Men may also be interested to get involved but may not know how to [6.2, P23]. To create an enabling environment, studies need to determine ‘how to engage communities using new ‘outside the box’ approaches’, especially with adolescents [6.5, P19].

An additional research gap which was highlighted by our review of preconception interventions but not identified in the stakeholder interviews was the replication of food supplementation trials, for example the Mumbai Maternal Nutrition Programme^9^ and Women first^8^ trials in India and Pakistan, across diverse contexts.^2^ Similarly, replication of studies from global contexts in South Asia may be beneficial, such as the PRECONCEPT micronutrient supplementation intervention in Vietnam which undertook longitudinal follow-up of women and their children.^24^

#### How to intervene using new approaches

Most research stakeholders suggested that randomised controlled trials are needed to determine ‘how effective interventions are’ [6.11, P08]. We need to research on ‘which components are required within complex interventions’ to address the drivers of malnutrition, guided by well thought-through theories of change [6.9, P10] and impact evaluation [6.9, P13].

Another under-explored aspect is to determine ‘what interventions communities want’ and involve adolescents and young people in ‘co-designing interventions’ based on what they think will work in their setting, to ensure that interventions are as impactful as possible [6.8, P23]. This is especially important in the context in which adolescent girls and young newlyweds often lack agency in their households.^25^

Calculating ‘the cost-benefit of preconception nutrition interventions’ was considered crucial to inform scale-up [6.10, P13] and to persuade governments to prioritise preconception programming. However, some stakeholders felt that a lack of motivation to improve women and girls’ nutrition, rather than a lack of funds, prevented progress [6.10, P14].

While some felt that the evidence base on what works is lacking, others expressed that we already know what works, and now the question is ‘how to translate research into practice’ using implementation research [6.7, P09]. This was also highlighted by our review of preconception interventions, which recommended implementation research to assess intervention feasibility, explore context specific barriers, and improve community awareness, to inform scale up.^2^

Lastly, research stakeholders raised the question of ‘how to harness collaborative networks to coordinate research’ to address priority research gaps, recommending the establishment of a South Asia collective [6.6 P19].

### Validating findings with the South Asia Preconception Nutrition Collective

Following a comprehensive review of the presented findings and stakeholder inputs, members of the South Asia Preconception Nutrition Collective (SAPNC) assembled key messages and recommendations for preconception nutrition.

Within the first high-level theme–understanding the preconception nutrition problem–SAPNC members emphasised the need to consider a broader range of nutritional deficiencies beyond anaemia, while also accounting for the impact of social determinants and mental health on preconception nutrition. To bridge critical data gaps, use of standardised methods and surveillance systems were highlighted as priorities for improving program design, delivery and monitoring.

Within the second high−level theme–how to intervene in preconception nutrition–SAPNC members advocated for a shift from women-centred approaches to interventions targeting couples and families. Outreach to couples should involve diverse modalities such as fixed-day or fixed-site services, integration with family planning programs and marriage registration systems or newlywed couple cards, and engagement via civil societies. Outreach mechanisms must be adapted to context, geography, and community needs. SAPNC members recommended research to support the development of robust mechanisms for registering and screening eligible couples, including a preconception screening tool and pathways to intervene at least 90 days before conception.^1^, ^2^, ^3^ Given the vast diversity within South Asia across countries, caste/ethnicity, wealth, rural/urban residence, and marital status, particularly when comparing across preconception life stages, outreach and interventions must be tailored to reflect the needs and realities of specific populations. Members also stressed the importance of understanding and overcoming community-level barriers and social norms which impact the uptake of interventions, reinforcing the call for formative research to inform SBC strategies. Rigorous process and impact evaluations are also called for to strengthen intervention delivery and facilitate a richer understanding of intervention impacts.

SAPNC members are also committed to establishing a South Asia research group on preconception nutrition to facilitate collaboration, consolidate a regional research agenda, harmonise research methodologies, generate evidence on life course impacts, and undertake multi-country studies and trials. Specific areas of research highlighted by SAPNC members included i) revisiting the preconception and pregnancy folic acid dosage guidelines, ii) comparing the effectiveness of MMS versus dietary approaches on women’s health and pregnancy outcomes, iii) comparing the impact of different types, dosage and length of supplementation of MMS for diverse groups, and iv) undertaking cost-benefit analyses and cost-effectiveness studies.

## Discussion

Through interviews with key stakeholders and integration of insights from research and policy reviews,^1^, ^2^, ^3^ we have identified two central challenges. The first challenge relates to defining the preconception period while balancing life course and life-stage approaches.^26^ This is critical amidst persistent micronutrient deficiencies and rising overweight prevalence among reproductive-age women.^1^ Several stakeholders emphasised the importance of including adolescents as part of the preconception population and associated programming within a life course framework, regardless of whether or not they intend to get pregnant.^22^ However, others expressed concerns around the appropriateness and acceptability of considering adolescents as preconceptual, suggesting that targeting resources to newlywed couples would represent a more acceptable and scalable approach to preconception care and nutrition. However, an approach addressing the nutritional status of married couples only erroneously suggests that the health of unmarried adolescents is less important than their married counterparts. Therefore, co-developed integrated approaches addressing adolescent^27^^,^^28^ and newlywed^29^ nutritional needs are required, through tailored, contextualised, life-stage specific programming that is practical and scalable. Participatory Learning and Action (PLA) approaches with adolescents both in and out of school have shown some potential in India,^7^^,^^30^ but may need to be coupled with poverty alleviation to enable adolescents to access the full benefits.^30^ Interventions may start through school−based education and nutrition support,^31^^,^^32^ although interventions need to be tailored for underweight adolescents in highly deprived areas.^33^ Such approaches may be integrated with approaches that engage newlywed couples, especially those of have had to leave school to marry.^34^ Establishing healthy habits in adolescence is a key enabler of lifelong healthy behaviours in adulthood, so starting interventions early is crucial,^35^ but more high quality studies are needed to inform intervention design.

The second challenge relates to how to intervene during preconception. The nutritional transition is particularly stark in South Asia as diets shift towards calorie-dense, micronutrient-poor, ultra-processed foods in favour of traditional wholegrains and vegetables, evidenced by increasing rates of non-communicable diseases (NCDs).^36^ Screening of nutritional status coupled with targeted supplementation amongst those found to be underweight and/or anaemic, plus dietary and lifestyle counselling for overweight or obese women/girls is called for.^37^ However the feasibility of nutritional screening and supplementation relies on existing government programmes for adolescents such as the Integrated Child Development Services (ICDS) in India.^38^ This may be harnessed to target preconception women and girls through systems strengthening interventions of Adolescent Health Days (AHDs), participation in which was associated with improved uptake IFA and deworming and lower odds of thinness.^7^ Moreover, the rapidly shifting nutritional landscape in South Asia necessitates a coordinated response, integrating social protection strategies with private sector and SBC approaches. Social protection strategies are essential in addressing food insecurity but must be nutrition-sensitive^39^ to promote preconception nutrition. Moreover, a rights-based approach to address inequalities in preconception nutritional status and inform the provision of services across the region is called for. Social protection strategies need to be able to target households with nutritionally vulnerable newlywed couples to improve access to nutritious foods during the preconception period, particularly in areas with high food insecurity. Such strategies must harness SBC approaches to ensure existing social protection programs are promoting equal distribution of resources and empowering women and girls,^1^^,^^2^^,^^39^ within a context where social norms favour men in intrahousehold food distribution.^40^^,^^41^ One intervention worked with federations of women’s self-help groups and adolescent groups using a PLA approach, successfully increasing service uptake, suggesting that community mobilisation can facilitate SBC.^7^ Involvement of the private sector is also critical to ensure food systems are able to deliver sustainable change and address market inequalities.^42^ Until now, there has been insufficient attention to the potential of public-private partnerships and food systems approaches to improve access to nutritious foods, as well as strategies for increasing consumer demand for healthier options.^43^ Furthermore, engagement with government systems is critical to ensure longevity of interventions and ensure supply. Two programs in India which engaged with government systems were effective in increasing uptake of services^7^^,^^23^ and should be explored further through further research.

Important research gaps remain in understanding how to effectively intervene during preconception to address potential causal pathways and prioritise interventions based on local contexts and existing evidence. This reflects the lack of implementation evidence on effective strategies over the past decade.^22^ While frameworks such as UNICEF’s framework on maternal and child nutrition^44^ and Partap’s conceptual model^45^ emphasise the need to address health, nutrition, social, and WASH pathways within immediate, underlying, and enabling contexts, most interventions have primarily focused on individual-level dietary improvement.^2^^,^^45^ This has severely limited our understanding of the broader pathways to impact.^2^ Our review of interventions^2^ found that the Women and Infants Integrated Interventions for Growth Study (WINGS) was the most effective intervention at reducing small-for-gestational-age and low birth weight.^37^ WINGS combined food and micronutrient supplementation with counselling, health and nutrition screening and treatment, psychosocial support, and WASH components in preconception. However, its intensive nature relied heavily on project employees, who observed food consumption daily, presents scalability challenges. Therefore, research to explore scalability of the intervention is needed. Communities, including young people, should be engaged in co-designing interventions that span multiple causal pathways to ensure relevance and impact,^46^^,^^47^ which should be costed to inform the scaling up of effective interventions.^48^

Furthermore, our review of preconception interventions in South Asia identified limited evidence regarding cultural barriers to preconception nutrition interventions, underscoring the necessity for qualitative formative research and process evaluation to unpick pathways to impact.^2^ Such research is critical to better understanding the cultural obstacles to optimal health and nutrition during preconception among men and women in South Asia, a region with distinct social norms that prioritise rapid conception after marriage, stigmatise infertility, and where open conversations about ‘trying to conceive’ may be considered embarrassing or inappropriate.^49^^,^^50^ Effective interventions should incorporate SBC strategies, alongside education, empowerment, and improved access to nutritious foods, leveraging platforms such as schools, workplaces, and community settings.^26^^,^^51^

Our review of policy and program mapping for each country revealed that nearly all countries are incorporating preconception nutrition into their maternal nutrition policies and implementing evidence-based interventions to enhance maternal and child health, in line with WHO and UNICEF guidelines.^3^ Despite most of the recommended preconception nutrition interventions being mentioned in the national policies and guidelines in South Asia, dedicated preconception nutrition programs are rare, necessitating advocacy for prioritisation.^3^ Lessons from countries like Sri Lanka, India, and Nepal offer insights into intervention delivery regionally. Collaborative networks across South Asia will be essential for coordinating research, advocating for focusing on preconception nutrition within existing programmes, supporting countries in designing interventions, and addressing systemic bottlenecks precluding progress on preconception nutrition in South Asia region.

This study engaged stakeholders actively involved in preconception nutrition research and programme implementation in South Asia, complemented by insights from global experts. While this approach ensured relevance to regional priorities, it may have excluded perspectives from frontline health workers, community organisations, and adolescents, who are critical actors in preconception nutrition. Additionally, we identified stakeholders from papers cited in reviews undertaken by the coauthor team^1^, ^2^, ^3^ and through existing professional networks, which may have inadvertently excluded key stakeholders. Moreover, more than half of the 64 researchers approached did not respond, no stakeholders from Afghanistan, the Maldives were identified, and those from Sri Lanka were unavailable, limiting the representativeness of our sample. The regional consultation in Delhi further refined our findings, and did involve a representative from Sri Lanka, but participant representation was influenced by logistical feasibility and availability of stakeholders. While validation in this consultation strengthened credibility, a follow-on to our study could involve broader engagement with diverse actors, including health practitioner associations, and a more structured ranking of research priorities using methodologies such as CHNRI^22^ or Delphi methods,^51^ which may improve the generalisability and applicability of these findings. However, our novel thematic analysis of stakeholder interview data enabled development of in-depth perspectives and provides a useful framework for grouping preconception priorities and selecting research questions as per variable contexts across the South Asian region.

## Conclusion

Addressing preconception nutrition in South Asia requires a multifaceted approach that considers the complexities of defining the preconception period and the challenges of effective interventions. Findings from our stakeholder interviews and regional reviews highlight the need for integrated, life-stage-specific strategies that include both adolescents and newlyweds to ensure sustained improvements in nutritional status. Existing interventions demonstrate the potential of combining direct nutrition interventions with community mobilisation and the importance of addressing health, nutrition psychosocial and WASH together. However, scalability remains a key challenge, necessitating stronger engagement with government systems and the private sector. Critical research gaps persist, particularly in understanding causal pathways, cultural barriers, and the broader social determinants of preconception nutrition. Future efforts must prioritise formative and implementation research to inform context-specific interventions across the life course and drive policy change. Strengthening regional collaborations and leveraging lessons from successful programmes across South Asia will be vital in scaling up effective strategies and ensuring long-term improvements in preconception nutrition.

## Contributors

Conceptualisation: VS, NS and FM.

Supervision and resources: VS and ZM.

Project Administration: NS, FM and VS.

Methodology: NS, FM and VS.

Investigation: FM and NS conducted interviews.

Data Curation: FM and NS checked, anonymised and corrected transcripts of interviews and curated the data.

Formal analysis: FM and NS.

Visualisation: FM and NS prepared the figures.

Validation: NS, FM, DS, RC, AH, JH, ZM and VS took part in a workshop in Delhi to Validate the findings from the qualitative data.

Writing—original draft: FM and NS wrote the first draft and prepared the final draft for submission.

Writing—review & editing: All authors (NS, FM, DS, RC, AH, JH, ZM and VS) read and commented and provided edits (where needed) on various drafts and approved the final manuscript.

## Data sharing statement

Anonymised transcripts can be made available by individual request.

## Declaration of interests

VS and ZM are employed by UNICEF Regional Office for South Asia which funded this study. Other than this, the authors declare no other conflicts of interest. Authors were not precluded from accessing data in the study, and they accept responsibility to submit for publication.

## References

[1] Miller F., Sethi V., Schoenaker D. (2025). Preconception malnutrition among women and girls in south Asia: prevalence, determinants, and association with pregnancy and birth outcomes. Lancet Reg Health Southeast Asia.

[2] Saville N.M., Dulal S., Miller F. (2025). Effects of preconception nutrition interventions on pregnancy and birth outcomes in south Asia: a systematic review. Lancet Reg Health Southeast Asia.

[3] Hazra A., Choedon T., Shrivastav M. (2025). Policies and programmes to improve preconception nutrition in south Asia. Lancet Reg Health Southeast Asia.

[4] Attride-Stirling J. (2001). Thematic networks: an analytic tool for qualitative research. Qual Res.

[5] WHO (2013).

[6] Stephenson J., Heslehurst N., Hall J. (2018). Before the beginning: nutrition and lifestyle in the preconception period and its importance for future health. Lancet.

[7] Kumar A., Sethi V., Wagt Ad (2023). Evaluation of impact of engaging federations of women groups to improve women’s nutrition interventions- before, during and after pregnancy in social and economically backward geographies: evidence from three eastern Indian States. PLoS One.

[8] Dhaded S.M., Hambidge K.M., Ali S.A. (2020). Preconception nutrition intervention improved birth length and reduced stunting and wasting in newborns in South Asia: the Women First Randomized Controlled Trial. PLoS One.

[9] Potdar R.D., Sahariah S.A., Gandhi M. (2014). Improving women’s diet quality preconceptionally and during gestation: effects on birth weight and prevalence of low birth weight--a randomized controlled efficacy trial in India (Mumbai Maternal Nutrition Project). Am J Clin Nutr.

[10] Rai R.K., Shinde S., De Neve J.W., Fawzi W.W. (2023). Predictors of incidence and remission of anemia among never-married adolescents aged 10-19 Years: a population-based prospective longitudinal study in India. Curr Dev Nutr.

[11] Chauhan S., Kumar P., Marbaniang S.P., Srivastava S., Patel R. (2022). Prevalence and predictors of anaemia among adolescents in Bihar and Uttar Pradesh, India. Sci Rep.

[12] Scott S., Lahiri A., Sethi V. (2022). Anaemia in Indians aged 10-19 years: prevalence, burden and associated factors at national and regional levels. Matern Child Nutr.

[13] Ford N.D., Bichha R.P., Parajuli K.R. (2022). Factors associated with anaemia among adolescent boys and girls 10-19 years old in Nepal. Matern Child Nutr.

[14] Habib M.A., Raynes-Greenow C., Soofi S.B. (2018). Prevalence and determinants of iron deficiency anemia among non-pregnant women of reproductive age in Pakistan. Asia Pac J Clin Nutr.

[15] Neha C., Purushottam K., Haripriya H. (2022). A study to assess the prevalence of anemia among patients attending an urban health training center in Bihar: one year experience of anemia Mukt Bharat test and treat campaign. Asian J Med Sci.

[16] Shanbhag D.N., Goud R., Ramesh N. (2016). Prevalence and correlates of anemia among mothers of children aged 0–23 months in three districts of Karnataka, India. Int J Med Sci Publ Health.

[17] West K.P., Katz J., Khatry S.K. (1999). Double blind, cluster randomised trial of low dose supplementation with vitamin A or beta carotene on mortality related to pregnancy in Nepal. The NNIPS-2 Study Group. BMJ.

[18] D’Souza N., Behere R.V., Patni B. (2021). Pre-conceptional maternal vitamin B12 supplementation improves offspring neurodevelopment at 2 Years of age: PRIYA trial. Front Pediatr.

[19] Khambalia A.Z., O’Connor D.L., Macarthur C., Dupuis A., Zlotkin S.H. (2009). Periconceptional iron supplementation does not reduce anemia or improve iron status among pregnant women in rural Bangladesh. Am J Clin Nutr.

[20] Young A.E., Kemp J.F., Uhlson C. (2021). Improved first trimester maternal iodine status with preconception supplementation: the Women First Trial. Matern Child Nutr.

[21] Ramakrishnan U. (2019). Nutrition education during the preconception period. Nestle Nutr Inst Workshop Ser.

[22] Dean S., Rudan I., Althabe F. (2013). Setting research priorities for preconception care in low- and middle-income countries: aiming to reduce maternal and child mortality and morbidity. PLoS Med.

[23] Doke P.P., Chutke P.A., Palkar H.S. (2024). Implementation of preconception care for preventing adverse pregnancy outcomes in rural and tribal areas of Nashik District, India. Prev Med Rep.

[24] Nguyen P.H., Young M., Gonzalez-Casanova I. (2016). Impact of preconception micronutrient supplementation on anemia and iron status during pregnancy and postpartum: a randomized controlled trial in rural Vietnam. PLoS One.

[25] Morrison J., Basnet M., Sharma N. (2023). Eating for honour: a cultural-ecological analysis of food behaviours among adolescent girls in the southern plains of Nepal. PLoS One.

[26] Hall J., Chawla M., Watson D. (2023). Addressing reproductive health needs across the life course: an integrated, community-based model combining contraception and preconception care. Lancet Public Health.

[27] Bhutta Zulfiqar A., Sharma Drishti, Shafique Sohana, Rashidi K. (2025). Improving adolescent health and nutrition in South Asia. BMJ.

[28] UNICEF (2025).

[29] Diamond-Smith N., Mitchell A., Cornell A. (2022). The development and feasibility of a group-based household-level intervention to improve preconception nutrition in Nawalparasi district of Nepal. BMC Public Health.

[30] Bhatia K., Rath S., Pradhan H. (2023). Effects of community youth teams facilitating participatory adolescent groups, youth leadership activities and livelihood promotion to improve school attendance, dietary diversity and mental health among adolescent girls in rural eastern India (JIAH trial): a cluster-randomised controlled trial. SSM Popul Health.

[31] Thakur J.S., Bharti B., Tripathy J.P., Dhawan V., Bhansali A. (2016). Impact of 20 Week lifestyle intervention package on anthropometric biochemical and behavioral characteristics of schoolchildren in north India. J Trop Pediatr.

[32] Leventhal K.S., DeMaria L.M., Gillham J.E., Andrew G., Peabody J., Leventhal S.M. (2016). A psychosocial resilience curriculum provides the “missing piece” to boost adolescent physical health: a randomized controlled trial of Girls First in India. Soc Sci Med.

[33] Rose-Clarke K., Pradhan H., Rath S. (2019). Adolescent girls’ health, nutrition and wellbeing in rural eastern India: a descriptive, cross-sectional community-based study. BMC Public Health.

[34] Marphatia A.A., Saville N.M., Amable G.S. (2019). How much education is needed to delay women’s age at marriage and first pregnancy?. Front Public Health.

[35] Hargreaves D., Mates E., Menon P. (2022). Strategies and interventions for healthy adolescent growth, nutrition, and development. Lancet.

[36] Vicziany M. (2021). The modernisation of South Asia’s disease burden: 1950 to 2021. South Asia J South Asian Stud.

[37] Taneja S., Chowdhury R., Dhabhai N. (2022). Impact of a package of health, nutrition, psychosocial support, and WaSH interventions delivered during preconception, pregnancy, and early childhood periods on birth outcomes and on linear growth at 24 months of age: factorial, individually randomised controlled trial. BMJ.

[38] Khapre M.P., Kishore S., Sharma A. (2019). Utilization of ICDS program by adolescent girls and implementation barriers in Urban Rishikesh, India. J Fam Med Prim Care.

[39] Scott S., Neupane S., Alderman H. (2023).

[40] Harris-Fry H., Shrestha N., Costello A., Saville N.M. (2017). Determinants of intra-household food allocation between adults in South Asia - a systematic review. Int J Equity Health.

[41] Harris-Fry H.A., Paudel P., Shrestha N. (2018). Status and determinants of intra-household food allocation in rural Nepal. Eur J Clin Nutr.

[42] Sun Business Network Leveraging public-private partnerships to champion better nutrition in South Asia 2019. https://sunbusinessnetwork.org/leveraging-public-private-partnerships-to-champion-better-nutrition-in-south-asia/.

[43] Alliance2015 (SmitMwanamwenge Marjolein) (2018). Global lessons learned and an overview of approaches of Alliance2015 partners.

[44] UNICEF (2021).

[45] Partap U., Chowdhury R., Taneja S. (2022). Preconception and periconception interventions to prevent low birth weight, small for gestational age and preterm birth: a systematic review and meta-analysis. BMJ Glob Health.

[46] Meloncelli N., O’Connor H., de Jersey S. (2024). Designing a behaviour change intervention using COM-B and the Behaviour Change Wheel: Co-designing the Healthy Gut Diet for preventing gestational diabetes. J Hum Nutr Diet.

[47] Knowles S., Sharma V., Fortune S., Wadman R., Churchill R., Hetrick S. (2022). Adapting a codesign process with young people to prioritize outcomes for a systematic review of interventions to prevent self-harm and suicide. Health Expect.

[48] Warren A.M., Frongillo E.A., Rawat R. (2020). Building implementation science in nutrition. Adv Nutr.

[49] Miller F.A., Dulal S., Rai A., Gram L., Harris-Fry H., Saville N.M. (2023). “Can’t live willingly”: a thematic synthesis of qualitative evidence exploring how early marriage and early pregnancy affect experiences of pregnancy in South Asia. PLoS Glob Public Health.

[50] Mitchell A., Puri M.C., Dahal M., Cornell A., Upadhyay U.D., Diamond-Smith N.G. (2023). Impact of Sumadhur intervention on fertility and family planning decision-making norms: a mixed methods study. Reprod Health.

[51] Boyle J.A., Black K., Dorney E. (2022). Setting preconception care priorities in Australia using a Delphi technique. Semin Reprod Med.

