# Supplementary material for: Bridging the gaps: advancing preconception nutrition in South Asia through evidence, policy, and action
Source: Lancet Reg Health Southeast Asia. 2025 Apr 24;36:100585. doi: 10.1016/j.lansea.2025.100585 (PMC12105509; doi:10.1016/j.lansea.2025.100585)
Supplement: Supplementary files S1–S5 [file mmc1.docx]

**Supplementary File for** **paper entitled “****Bridging the gaps: Advancing Preconception Nutrition in South Asia through Evidence, Policy, and Action”**

By Faith Miller, Vani Sethi, Avishek Hazra, Danielle Schoenaker, Ranadip Chowdhury, Jane Hirst, Zivai Murira, Naomi M Saville

**Table of contents**

[1. Supplementary File S1. List of semi-structured interview participants who gave permission to be named 2](#_Toc193118452)

[2. Supplementary File S2. Participants at the Delhi Meeting of the South Asian Preconception Nutrition Collective (SAPNC) held 21-22 November 2024 entitled “Evidence Synthesis on Preconception Nutrition in South Asia and Way Forward” 3](#_Toc193118453)

[3. Supplementary File S3. Topic Guides 5](#_Toc193118454)

[4. Supplementary File S4. Coding framework and density of codes from qualitative analysis of stakeholder interviews. 8](#_Toc193118455)

[5. Supplementary File S5. Supporting evidence for the basic themes of the thematic network 10](#_Toc193118456)

# **Supplementary File S1. List of semi-structured interview participants who gave permission to be named**

| SN | Respondent type | Name | Country of research | Project or publication | Affiliation |
| --- | --- | --- | --- | --- | --- |
| 1 | Researcher | Parul Christian | Nepal, Bangladesh | JVitA trials in Bangladesh / Experience with Bill and Melinda Gates Foundation | Johns Hopkins University (JHU) |
| 2 | Researcher | Malay K. Mridha | Bangladesh | Rang-Din Nutrition Study (RDNS) trial, Bangladesh | BRAC (Bangladesh Rural Advancement Committee) University James P Grant School of Public Health, Dhaka, Bangladesh |
| 3 | Researcher | Ranadip Chowdhury | India | Women and Infants Integrated Interventions for Growth Study (WINGS) trial investigator, | Society for Applied Studies, New Delhi |
| 4 | Researcher | Sangappa M. Dhaded | India | Investigator on Women First Trial, India | Jawaharlal Nehru Medical College, Belgaum, India |
| 5 | Researcher | Hema Diwakar | India | International Federation of Gynaecology and Obstetrics (FIGO) and Federation of Obstetric and Gynaecological Societies of India (FOGSI), India | Gynaecologist, Obstetrician, Member of FIGO Executive Board |
| 6 | Researcher | Prakash Doke | India | Healthy Parents Healthy Child Initiative | Community Medicine Department, Bharati Vidyapeeth (Deemed to be University) (BVDU) Medical College, Pune, India |
| 7 | Researcher | Mark Hanson | UK, Globally, Involved in HeLTI study in India. | Preconception Lancet commission / previous co-chair of International Federation of Gynaecology and Obstetrics (FIGO) | University of Southampton, UK |
| 8 | Researcher | Manisha Nair | India | UK-India collaborative platform for maternal and perinatal health research (MaatHRI) | University of Oxford, UK |
| 9 | Researcher | Reedhika Puliani | India | Preconception scoping review author /PhD student | MS Ramaiah University of Applied Sciences, Bangalore, India |
| 10 | Researcher | Anonymous | India | NA | NA |
| 11 | Researcher | Joanne Katz | Nepal | Nepal Nutrition Intervention Project Sarlahi (NNIPS) | Johns Hopkins University (JHU), USA |
| 12 | Researcher | Naomi Saville | Nepal | Principal investigator of the Low Birthweight South Asia Trial (LBWSAT) / Author of UNICEF preconception reviews | University College London, UK |
| 13 | Researcher | James Tielsch | Nepal | Nepal Nutrition Innovation Project Sarlahi (NNIPS) | George Washington University, USA |
| 14 | Researcher | Keith West | Nepal, Bangladesh | JVitA trials in Bangladesh / Nepal Nutrition Intervention Project Sarlahi (NNIPS) | Johns Hopkins University (JHU), USA |
| 15 | Researcher | Anonymous | Nepal | Director, Nepal Nutrition Intervention Project Sarlahi (NNIPS) | Johns Hopkins University (JHU), USA |
| 16 | Researcher | Sajid Bashir Soofi | Pakistan | Matiari emPowerment and Preconception Supplementation (MaPPS) Trial | Aga Khan University, Pakistan |
| 17 | Researcher | Zulfiqar A. Bhutta | Pakistan | Matiari emPowerment and Preconception Supplementation (MaPPS) Trial | Aga Khan University, Pakistan |
| 18 | Researcher | Jo-Anna Baxter | Pakistan | Matiari emPowerment and Preconception Supplementation (MaPPS) Trial | University of Toronto, Canada |
| 19 | Researcher | Anonymous | Vietnam | NA | NA |
| 20 | Researcher | Judith Stephenson | UK / Globally | Preconception Lancet series | University College London, UK |
| 21 | Programme implementer | Aniruddha Deshpande | India | Investigator on Healthy Parents Healthy Child Initiative | Assistant Director of Health Services, Government of Maharashtra, India |
| 22 | Programme implementer | Manoj Pal | India | Involved in scale up of the WINGS trial interventions | Engender Health, India |
| 23 | Programme implementer | Anonymous | Bhutan | NA | NA |

Note: Three stakeholders (from India, Bhutan and Vietnam) preferred to remain completely anonymous and two (involved in studies in Nepal and Pakistan) wanted only their role but not their name to be mentioned. NA means information not available.

# **Supplementary File S2. Participants at the Delhi Meeting of the South Asian Preconception Nutrition Collective (SAPNC) held 21-22 November 2024 entitled “Evidence Synthesis on Preconception Nutrition in South Asia and Way Forward”**

| **SN** | **Name** | **Designation** | **Organization** |
| --- | --- | --- | --- |
| **International Delegates** | | | |
| 1 | Jane Hirst | Chair Women's Health | George Institute of Global Health, UK |
| 2 | Anju Aggarwal | Government Engagement Advisor | The George Institute of Global Health, India |
| 3 | Naomi Saville | Principal Research Fellow | University College London Consulting, UK |
| 4 | Tsering P. Lama | Associate, International Health (Affiliated) | JHU-Nepal / Nepal Nutrition Intervention Project Sarlahi (NNIPS) |
| 5 | Pemba Yangchen | Dy. Chief Program Officer | Centre of Nutrition, Bhutan |
| 6 | Sanjeeva Godakandange | Consultant Community Physician | Government of Sri Lanka |
| **Indian Delegates** | | | |
| 7 | Kalyanaraman Kumaran | Clinical Scientist/Senior Lecturer | University of Southampton, UK |
| 8 | C.S. Yajnik | Director, Diabetes Unit and MRC Lifecourse Epidemiology Unit | KEM Hospital, Pune, India |
| 9 | Prakash P. Doke | Professor | BVDU, Medical College, Pune, India |
| 10 | Sangappa Dhaded | Professor of Neonatology | JN Medical College, Belagavi, India |
| 11 | Sundarnag Ganjekar | Professor (Associate) | NIMHANS, Bangalore, India |
| 12 | Biraj Swain | CM Chair Professor | National Law University, Orissa, India |
| 13 | Rashmi Ranjan Das | Professor, Department of Paediatrics | AIIMS, Bhubaneshwar, India |
| 14 | Dharitri Swain | Assistant Professor | AIIMS, Bhubaneshwar, India |
| **Based out of Delhi** | | | |
| 15 | Neena Bhatia | Professor, Food and Nutrition and Food Technology | Lady Irwin College, New Delhi, India |
| 16 | Divya Tripathi | Assistant Professor | Manav Rachna International Institute of Research and Studies, Faridabad, India |
| 17 | Reema Mukherjee | Scientist | ICMR, New Delhi, India |
| 18 | Priyanka Bansal | Scientist | ICMR, New Delhi, India |
| 19 | Manju Puri | Director Professor | Lady Hardinge Medical College, New Delhi, India |
| 20 | Praveen Kumar | Director of Professor | Kalawati Saran Children’s Hospital, New Delhi, India |
| 21 | Sarita Anand | Associate Professor, Dept. of Development Communication and Extension, Lead | ROSHNI, New Delhi, India |
| 22 | Ranadip Chowdhury | Scientist | SAS, New Delhi, India |
| 23 | Raj Verma | Program Officer II | Population Council, New Delhi, India |
| 24 | Avishek Hazra | Director, Program and Policy Evaluations | Population Council Consulting, New Delhi, India |
| 25 | Monica Srivastava | Specialist, Program and Policy Research | Population Council Consulting, New Delhi, India |
| 26 | Tashi Choedon | Consultant | IEG, New Delhi, India |
| 27 | Shilpi Nain | Professor | Lady Hardinge Medical College, New Delhi, India |
| **UNICEF** | |  |  |
| 28 | Zivai Murira | Advisor, Nutrition | UNICEF, ROSA |
| 29 | Vani Sethi | Nutrition Specialist | UNICEF, ROSA |
| 30 | Aryan de Wagt | Deputy Representative-Programmes | UNICEF, India |
| 31 | Marie-Claude Desilets | Chief, Nutrition | UNICEF, India |
| 32 | Sylvie Chamois | Nutrition Specialist | UNICEF, India |
| 33 | Preetu Mishra | Nutrition Specialist | UNICEF, India |
| 34 | Richa Pandey | Nutrition Specialist | UNICEF, India |
| 35 | Praween Agrawal | Research Officer | UNICEF, India |
| 36 | Lopamudra Tripathy | SBC Specialist | UNICEF, India |
| 37 | Sameer Pawar | Nutrition Specialist | UNICEF, India |
| 38 | Prafulla Ranjan Mishra | Nutrition Officer | UNICEF, India |
| 39 | Smita Sinha | Program Associate | UNICEF, India |
| 40 | Khyati Vats | Consultant | UNICEF, India |
| **Online Attendees Nourishing South Asia: Regional Technical Consultation** | | | |
| **SN** | **Name** | **Designation** | **Country** |
| 41 | Faith Miller | Post Graduate Teaching Assistant, University College, London | UK |
| 42 | Manisha Nair | Associate Professor, Epidemiologist, University of Oxford |  |
| 43 | Danielle Schoenaker | Senior Research Fellow, University of Southampton |  |
| 44 | Nethanjalie Mapitigamaa | Consultant Community Physician, Govt of Sri Lanka | Sri Lanka |
| 45 | Dinusha Perera | National Programme Manager, Govt of Sri Lanka |  |
| 46 | Abner Daniel # | Health and Nutrition Manager, UNICEF |  |
| 47 | Dhammica Rowel | Nutrition Specialist, UNICEF |  |
| 48 | Asheber Gaymn # | Nutrition Specialist, UNICEF | Nepal |
| 49 | Naveen Paudel # | Nutrition Officer, UNICEF |  |
| 50 | Hannah Gardener | Consultant |  |
| 51 | Ankita Mondal | Consultant |  |
| 52 | Naureen Arshad # | Nutrition Officer, UNICEF | Pakistan |
| 53 | Bidhan Krishna Sarker  (online) | Associate Scientist, Maternal and Neonatal Health, ICDDR Bangladesh | Bangladesh |
| 54 | Ireen Akhter Chowdhury # | Nutrition Officer, UNICEF |  |
| 55 | Mohd. Aziz # | Nutrition Officer, UNICEF |  |
| 56 | Ahmadwali Aminee # | Nutrition Specialist, UNICEF | Afghanistan |
| 57 | Mohd. Ameen # | Nutrition Specialist, UNICEF |  |
| 58 | Kinley Dorji # | Nutrition Officer, UNICEF | Bhutan |
| 59 | Indrani Chakma^#^ | Health & Nutrition Specialist, UNICEF |  |
| 60 | Dr Sushree Sangita | Lady Irwin College, New Delhi | India |
| 61 | Usha Sriram | Physician, Endocrinologist and Diabetes Specialist, DIWAS |  |
| 62 | Preety Rajbanshi | Senior Research Fellow, The George Institute for Global Health |  |

# indicates 10 participants that joined only briefly online

# **Supplementary File S3. Topic Guides**

Topic guides used to conduct the stakeholder interview are provided below. The questions to programme implementers were slightly different from those asked to researchers.

**Topic guide for preconception researchers**

Instructions to start:

Check that the participant has read the information sheet and signed the consent form before starting the interview.

The topic guide provides main numbered questions and sub-questions which can be used as probes as needed, depending on the detail provided in the main answer.

Introduce yourself to the respondent:

“Hello, my name is Naomi Saville, and I am a Principal Research Associate at the University College Institute for Global Health. I am based in Nepal where I undertake research on maternal and child nutrition. Thank you very much for giving up your time to talk to me today.

Currently my colleague Faith Miller and I are working with Vani Sethi and Zivai Murira at the UNICEF Regional Office for South Asia (ROSA) to collate evidence on preconception nutritional status, interventions that have been tested to improve preconception nutrition and to identify research gaps. We have recently reviewed the literature on studies testing the impact of preconception nutrition interventions upon birth outcomes and maternal nutrition during pregnancy in South Asia. This has shown that there are only a small number of studies which have tested the impact of preconception nutrition interventions and that there is a large gap in the evidence. So, in order to **identify the research gaps on the intervention packages for preconception nutrition care in South Asia which are impeding policy and programme action**, we would like to talk to you today about your experience in conducting research on the preconception period and your perceptions of research priorities for South Asia going forwards.

If you permit, we would like to record and transcribe this session so that we can analyse the discussion and compare and compile your perceptions with those of others working in the field. Unless you instruct us otherwise, we will remove your name and the name of others from the transcripts and not attach your name to any of the quotes that we extract from data. However, if there is something you want to say which you would *prefer* to be quoted by name you can tell us, and we may use your name for those statements is appropriate. Is it ok if I start the recording now?

1. I read with interest the paper[s] describing your study on [*add summary of the person’s study*]. Please can you tell me a little about that study, your experience in implementing it and its findings?
   1. How do you think the findings of your study could be used to inform policy and programmes in South Asia?
2. What do you think are the main nutritional challenges facing women and girls in the preconception period in your research setting?
   1. To what extent are these challenges common or different across the South Asia region?
3. The preconceptual period can be defined in several ways. What do you think is the best way to characterise the preconception period from the point of view of programme targeting?
   1. How might we reach girls or women who are soon to get married, recently married, and those who are between pregnancies?
   2. Do you have any ideas on how to target men in the preconception period?
   3. How should interventions differ if they are targeting adolescents as opposed to intervention targeting adults over 18 years?
4. What do you think are the priority research questions to be answered on preconception nutrition in South Asia?
5. What preconception nutrition intervention interventions do you think need testing in South Asia? Please can you describe the
   1. target group
   2. type of intervention (e.g. Social and Behaviour Change, micronutrient supplementation, supplementation with Specialised Nutritious Foods or with general food rations or other)
   3. intervention delivery mechanism
   4. type of research that should be undertake (e.g. cluster or individual RCT, longitudinal cohort or other designs)
6. How could these preconception interventions be integrated as programmes within national health, education or social protection systems?
7. What are the main policy issues affecting roll-out of preconception interventions in your setting?
   1. To what extent are these issues specific to your setting or generalisable across the region?
8. Do you have any more ideas or things you would like to say about the research gaps on the intervention packages for preconception nutrition care which are impeding policy and programme action in South Asia?

That is all the questions I had for you today. Thank you very much for your time and your valuable ideas.

**Topic guide for preconception programme implementers**

Instructions to start:

Check that the participant has read the information sheet and signed the consent form before starting the interview.

The topic guide provides main numbered questions and sub-questions which can be used as probes as needed, depending on the detail provided in the main answer.

Introduce yourself to the respondent:

Either:

“Hello, my name is Naomi Saville, and I am a Principal Research Associate at the University College Institute for Global Health. I am based in Nepal where I undertake research on maternal and child nutrition.”

Or

“Hello, my name is Faith Miller, and I am a Principal Research Associate at the University College Institute for Global Health. I am based in London where I undertake research on health and nutrition of adolescents, mothers, and children.”

“Thank you very much for giving up your time to talk to me today.

Currently my colleague and I are working with Vani Sethi and Zivai Murira at the UNICEF Regional Office for South Asia (ROSA) to collate evidence on preconception nutritional status, interventions that have been tested to improve preconception nutrition and identify research gaps. We have recently reviewed the literature on studies testing the impact of preconception nutrition interventions upon birth outcomes and maternal nutrition during pregnancy in South Asia. This has shown that there are only a small number of studies which have tested the impact of preconception nutrition interventions and that there is a large gap in the evidence. So, in order to **identify the research gaps on the intervention packages for preconception nutrition care in South Asia which are impeding policy and programme action**, we would like to talk to you today about your experience in conducting research on the preconception period and your perceptions of research priorities for South Asia going forwards.

If you permit, we would like to record and transcribe this session so that we can analyse the discussion and compare and compile your perceptions with those of others working in the field. Unless you instruct us otherwise, we will remove your name and the name of others from the transcripts and not attach your name to any of the quotes that we extract from data. However, if there is something you want to say which you would *prefer* to be quoted by name you can tell us, and we may use your name for those statements is appropriate. Is it ok if I start the recording now?

1. Please can you tell me a little about that preconception nutrition intervention programme, your experience in implementing it, its challenges and successes?
   1. How do you think the experiences from your programme could be used in policy and programmes in South Asia?
2. What do you think are the main nutritional challenges facing women and girls in the preconception period in your programme setting?
   1. To what extent are these challenges common or different across the South Asia region?
3. The preconceptual period can be defined in several ways. What do you think is the best way to characterise the preconception period from the point of view of programme targeting?
   1. How might we reach girls or women who are soon to get married, recently married, and those who are between pregnancies?
   2. Do you have any ideas on how to target men and boys in the preconception period?
   3. How should interventions differ if they are targeting adolescents as opposed to intervention targeting adults over 18 years?
4. What do you think are the priority research questions to be answered on preconception nutrition in South Asia?
5. What preconception nutrition intervention interventions do you think need testing in South Asia? Please can you describe the
   1. target group
   2. type of intervention (e.g. Social and Behaviour Change, micronutrient supplementation, supplementation with Specialised Nutritious Foods or with general food rations or other)
   3. intervention delivery mechanism
   4. type of research that should be undertake (e.g. cluster or individual RCT, longitudinal cohort or other designs)
6. How could these preconception interventions be integrated as programmes within national health, education or social protection systems?
7. What are the main policy issues affecting roll-out of preconception interventions in your setting?
   1. To what extent are these issues specific to your setting or generalisable across the region?
8. Do you have any more ideas or things you would like to say about the research gaps on the intervention packages for preconception nutrition care which are impeding policy and programme action in South Asia?

That is all the questions I had for you today. Thank you very much for your time and your valuable ideas.

# **Supplementary File S4. Coding framework and density of codes from qualitative analysis of stakeholder interviews.**

To code stakeholder interview transcripts an initial coding framework was developed inductively as informed by two reviews and an initial read-through of the first five transcripts. This framework was then applied to the remaining transcripts and updated iteratively through discussion between Faith Miller (FM) and Naomi Saville (NS).

| **Broad theme** | **Title of code** | **No of times coded** |
| --- | --- | --- |
| Nutritional challenges | Dietary choices / poor diets | 18 |
|  | Overweight | 14 |
|  | Underweight | 13 |
|  | Micronutrient deficiencies | 11 |
|  | Anaemia | 10 |
|  | Short stature | 2 |
| Who to target for preconception | Adolescents | 63 |
|  | Targeting men | 42 |
|  | Newly married | 40 |
|  | Lifecourse | 29 |
|  | Interpregnancy | 25 |
|  | Opposition to 'preconception' | 18 |
|  | Differences by age of married women | 13 |
|  | Unmarried | 11 |
| Drivers of poor nutrition | Social norms re: nutrition | 16 |
|  | Cost of food | 13 |
|  | Junk food | 10 |
|  | Age at marriage/pregnancy | 8 |
|  | Food Availability/Market functionality | 8 |
|  | Education/awareness of foods | 6 |
|  | Food Access | 6 |
|  | Climate or climate change | 5 |
|  | Other | 4 |
|  | Food Environment/system | 3 |
|  | Seasonality | 3 |
|  | Perceptions of medical interventions | 2 |
| Preconception work described | Micronutrient Supplementation trials | 22 |
|  | Food Supplementation trials | 21 |
|  | Describing preconception diets | 5 |
|  | Describing preconception nutritional status | 5 |
|  | Food fortification | 2 |
|  | Studies in industries | 1 |
|  | SBC studies | 0 |
| Priority research questions | Measuring nutritional status | 28 |
|  | Micronutrient supplementation | 27 |
|  | Food Supplementation | 26 |
|  | Diets | 21 |
|  | Targeting | 17 |
|  | Formative research on what preconception means to women | 15 |
|  | Other research topics | 15 |
|  | Social & Behaviour Change | 14 |
|  | Changing needs with nutritional transition | 12 |
|  | Cost-effectiveness | 9 |
|  | Social factors or inclusion | 9 |
|  | Leveraging existing programmes | 7 |
|  | Social Protection | 7 |
|  | Studies on delivery mechanism | 6 |
|  | Need to collate or aggregate studies | 4 |
|  | Understanding drivers of malnutrition | 4 |
|  | Policy | 3 |
| Type of intervention | SBC | 38 |
|  | Food supplementation | 36 |
|  | Micronutrient supplementation | 31 |
|  | Education materials | 26 |
|  | Family planning & preconception | 15 |
|  | Home diets | 11 |
|  | Other | 10 |
|  | Delaying age at marriage | 6 |
|  | School meals | 4 |
|  | Social protection | 3 |
|  | Gender-based violence | 2 |
| Research design | Cluster RCT | 13 |
|  | Individual RCT | 6 |
|  | Longitudinal cohort | 6 |
|  | Implementation research | 3 |
|  | Collate data from health systems | 1 |
|  | Improved monitoring of existing systems | 1 |
|  | Staged implementation (stepped wedge) | 1 |
| Targeting/delivery mechanism | Community level delivery | 65 |
|  | School level delivery | 38 |
|  | Health facility level delivery | 26 |
|  | Social media | 23 |
|  | Workplace delivery platform | 14 |
|  | Private sector delivery | 10 |
|  | Leverage existing program | 9 |
|  | Other | 8 |
| Implementation platform | Integrate across platforms | 27 |
|  | Education system | 26 |
|  | Health system | 19 |
|  | Social protection systems | 15 |
|  | Women and Child Development (WCD) ministry | 4 |
|  | Other | 3 |
| Issues affecting implementation | Research gaps | 39 |
|  | Lack of awareness amongst policy makers | 28 |
|  | Cost/lack of funding | 19 |
|  | Lack of awareness amongst reproductive age women | 17 |
|  | Government motivation | 16 |
|  | Lack of awareness amongst care providers | 14 |
|  | Silos / Lack of integration | 7 |
|  | Supply chain issues | 6 |
|  | Decentralised decision-making | 4 |
| Preconception policy | Lack of policy on preconception | 16 |
|  | Example of policies by setting | 12 |
|  | Policy to improve awareness of care providers | 5 |
|  | Policy to improve awareness of policy makers | 2 |
|  | Policy to improve awareness of repro age women | 2 |
|  | Private sector | 2 |
| Generalisability | Factors unique to specific settings | 31 |
|  | Factors common across South Asia | 20 |
|  | Rural vs urban considerations | 4 |

# **Supplementary File S5. Supporting evidence for the basic themes of the thematic network**

| Global theme | Organising theme | Basic theme new | Quote 1 | Quote 2 | Quote 3 | Quote 4 | Quote 5 | Quote 6 |
| --- | --- | --- | --- | --- | --- | --- | --- | --- |
| Understanding the preconception problem | Challenges in defining preconception | Blanket versus targeted interventions | say that the nutrition is going to be more impactful in a particular mothers with these ABCD characteristics, then we should try to look at those characteristics and try to select the population. so that you cover a larger number of population in a given budget, or whatever that you have, because, as I said, ultimately the cost is also is important as far as any intervention is concerned. | what supplement composition you would even choose would really depend on context, because you want to do some background assessment to see what are the micronutrient deficiencies. And you want to tailor, in my opinion, you wanna tailor your supplement to what's actually there. If iron deficiency is not actually an issue, then you probably don't wanna give people iron, cause that can have really big side effects and that can sort of undermine your entire supplementation regimen. |  |  |  |  |
|  |  | Importance and challenges considering adolescents as preconception | So, I mean, one of the things that to me one of the things that to me seems problematic is what does preconception mean? Umm, you know, is it sort of just periconceptional or is it sort of having an adolescent who goes into pregnancy as a young adult, sort of in good shape? | The third problem comes with adolescents is around anything around, consent etcetera, because they would even if we have to do an intervention, then the legality portion comes in. I mean not below 18. You have to at least tell the parents, or you have to keep parents in loop.  Whereas uh young adults would be more independent to listen to take that decision. | this immediately raised antibodies in some colleagues, actually even people in WHO who said, oh, you really should not be talking to young people about becoming parents and their children. You know, we don't want them to think about that. | And the thing we realized very quickly was that actually, health didn't really resonate with them at all.  I was seeing nutrition and exercise were 2 of the major things that we were talking to them about.  And it wasn't really until we began to talk to them in terms of an investment for the future. You want to have, you know, think in the same way that you know you want to get a flat, possibly you wanna get a car, you want in your married home to have white goods, and you know these people work incredibly hard. | If I was a young girl, I wouldn't want people to come to me and treat me like a walking, talking uterus. Which is what preconception implies. | I would say to you that even though the term is obvious, I would take conception out of it. |
|  |  |  | in my work has been in rural areas where girls get married at 16 to 17, they're pregnant by 17 and 18, they have a baby by 18 or 19 and the time between marriage and conception is frightfully short. And so that brings in when and how the and for what reason. There should be early adolescent interventions or before in order to better prepare women and neglected men as well for a preconception health that can lead to healthier pregnancy. | And then there's there's the sort of period of adolescence when when girls may not yet be married, they may still be in school, or they may be out of school because of having been excluded from school. But they're not yet married. But that's when they're they're nutritional status is already beginning to fail, and their their suffering with their anaemia or or low BMI or other micronutrient deficiencies. So, there's that, that's a different window again and that would, I think require either school or out of school programs, which are nutritional based programs | And so we hit adolescence and we've got an opportunity for that late adolescent growth spurt. And the evidence that we have, at least from some of the big national surveys in India, suggests that, umm, those kids are not realizing the full potential of an adolescent growth spurt, especially the girls. So, you know, they become stunted women of reproductive ag | One, because that's a very important stage, which actually sets a trajectory for their own health. So, I think rather than just see them as a preconceptional target audience, you know, I think they're a very important group because that's when we can really change trajectories then. You know, I think very early part of life. | So, adolescents like 15 to 19 years adolescents who are married in Bangladesh, 6% of them are currently pregnant. This is very high.... So, it also has an effect of her own nutritional status, not only just just the baby so, but like for adult we do not need to delay this first birth. So, delaying the first birth can be an intervention for adolescents | we didn't use preconception care as a term in that even once. And it wasn't by happenstance. It was deliberate. Yeah, because I felt very strongly that, you know, we should not really couch all at least young girls as mothers in waiting. |
|  |  | Improving men’s preconception health | Culturally it's not very common for the men or the husbands to get involved in the care of their wives during pregnancy or in the care of the children when they are very young. You know, that's mostly done by the mother and the grandmother, but but we actually discovered that many husbands wanted to get involved. And it was just a question of them not being able to know how to get involved so that that that's resulted in us developing 2 specific modules for husbands. So, one that focuses on their own health, but also one that focuses on how they can support their voices. | Because you know that initially the gender inequality, gender inequality.  Now I think the boys and men all suffering.  Whenever you talk about the gender, gender mean is actually the of the woman it's the the female issues.  So, it's it's not the man, but I think we need to support both the gender also | The husband and the senior leaders in the family in laws, mother in law, sister in law, they are having a very important say in the family decision | But you know, like, worldwide I mean sperm quality is declining on the declined so then? Also like infertility problems among men. This is also on the rise, so in that perspective. So, like I mean, there can be interventions as well say for example. One of the reasons which is affecting spawn [sperm] quality is zinc deficiency, so I and also like we do not know any idea about the nutrient status of men in our country or adolescent boys in our country. So, there can be like more research in that area and also target | I think any engagement of men in that kind of care has got to be much more holistic than just a narrow health and nutrition focus. I generally find that it's easier to engage men, particularly those who are fresh, would-be parents or newlyweds, in just general importance of preconception care for family health. And particularly they have generally reasonable stakes in the well-being of their own children. |  |
|  |  | Lifecourse approach considering population preconception | So, I mean, preconception goes all the way back to fetal life, right? And so when I think of, you know, in South Asia, when one thinks of preconception, one thinks of adolescence almost entirely right | One, because that's a very important stage, which actually sets a trajectory for their own health. So, I think rather than just see them as a preconceptional target audience, you know, I think they're a very important group because that's when we can really change trajectories then. You know, I think very early part of life. | the adolescent period has probably 2 important aspects for the health of their own selves rather than thinking about future generations.  We have done some work with adolescents and I think for behaviour change interventions particularly adolescence might be a good time to start those interventions because if, if, if, if some of those can be established at that age, then I think there's a chance it will actually carry on through.  And if at some point in the future they decide to have children, then that's absolutely fine | Because arguably, a woman's preconception period is the whole of our reproductive life. And that's not very good. So, I don't think anybody else yet has come up with an alternative, but I mean one has to divide I think one's thinking about the terminology between trying to help parents of tomorrow, let's say both men and women, I should say, women and men, and how one talks about that from what one might put into policy documents and talking to policymakers and practitioners and governments who would understand preconception? | let's get everybody nourished between 15 to 50. Umm, I think that and from program targeting I think that will be that won't be difficult. | The Who is going to come round to because the push back is is is this general population approach OK to be telling people who definitely want to conceive, but you shouldn't be going around telling everyone now, I don't really agree with that. I think it's the way you do it, but that's the point. If you do it within a life course framework, programmatic entry points which might be in different services, you can have those conversations etcetera and obviously avoiding all this sort of guilt tripping kind of stuff, |
|  |  | Preconception intervention duration depends on intervention | I feel like the pre/periconceptional period. And this is based mostly on, like, you know, fully deficiency and neural tube defects risk. I think the 3 months pre and 1st 3 months of the you know, embryonic period. 1st trimester seems like a good window. But, as we were discussing previously, I don't know if a 3 month intervention will get them to a nutritional plane which it is sufficient. But is that if that period is sufficient? And so I think in the studies we do in the interventions. That we test not in a programmatic context, there's still a knowledge gap to fill and perhaps doing something even that is status linked. | Now the other is maybe the what is the optimal duration? This is the one trial maybe we need to robust at least the Research on this issue about the preconceptionally, what, how would you define the preconception? Is it the three month, six month, after marriage, before marriage? | Do we need 12 months?  Do we need 18 months?  We just don't know yet. | You know, especially if it's a behaviour change intervention you know, is it enough to intervene six months before they become pregnant? | And we put it in three ways. There's the sort of biology, biological, which is to do with folic acid. And you've got to, and that's quite so that's quite that's weeks before conception. That's if you like the biological perspective, then there's a very important one which we call the individual perspective is whenever you decide I want to get pregnant,  we want a baby that's your conscious. That's the start of your conscious preconception period. | A lot of the health risks or things like overweight and obesity, you can't shift easily, so you need more time if nothing else to sort of. I mean, some people can stop smoking or change their diet very quickly, but for many people they can't. And also you can't shed lots of weight very quickly for sure. So, we talked about a public health perspective, which would be, , more like years before conception, conceptually. |
|  |  | Pregnancy planning approach at individual level | And I guess the depth of material, so you could start telling people about concepts when they're younger, but then get it more, once it becomes more real to you. And we're like, it's in your future, then you get a bit into more depth, but at least you've sort of primed someone. I guess they're a bit aware of the concept from earlier on, and I guess when you're younger you probably want a bit more about maintaining good health in your own health and well-being and how to have appropriate nutritional intake. Versus when you're a bit older it's more about planning to have a baby and what should you do in advance of becoming pregnant? What are? What are things that you can do? | I know there's a big shift towards adolescent girls and improving their nutrition, which is kind of preconception, but not targeted preconception per se. It's sort of improving the health and well-being. | But we have very we actually significant effect on low birth weight as per protocol analysis, we are as per protocol analysis this in which the mother received exactly supplementation 6 month prior to pregnancy.  So, that's why it's it's very important.  You need to work on your adolescent girl and entry conception phase. |  |  |  |
|  | Research gaps to understand preconception burden | Formative research on what preconception means to women is needed | So, we have already done two years of formative work where we worked with the local communities to sort of get their perspectives on the study, what their needs and priorities were | The the second thing about adolescence, I think is it's it's very important to work with them to actually get them to co-develop the interventions with us. And that's something again we have discovered through some of the formative work. You know, actually did a study on adolescent | cannot tell you why somebody who is a daily wage worker would prefer to go to to, you know, earn his daily wage rather than to spend that time and come and get supplements or get tested.  That won't be his priority, I'm guessing, or her priority. For me yes, I would thi | I think it's very really important to start engaging the young people themselves with the question and try and think how you frame it | That's why the life course approach, even though I don't listen, there are so many 20 - 25% obesity. Suddenly they come to preconception. And you say you optimize your weight, you do this. That and the other they get psychologically upset. That's all that happens, because they can't, they say behaviour change issue, so they can't. |  |
|  |  | Multiple micronutrient deficiencies must be addressed | We know that like even the latest uh micronutrient survey identified very well that like there is a high level of micronutrient deficiency among non pregnant nonlactating women in Bangladesh like vitamin D deficiency is 70%, B12 20% folate deficiency is 29%, zinc is 43% anaemia 30%, iodine deficiency 30% despite these universal solitaire [salt] iodisation program. So, of course, like in terms of preconception nutrition, this micronutrient deficiency, especially those micronutrients that are responsible for the well being of both women and children. Uh, so that is that deficiency is is a is a challenge like the high level deficienc | Yeah, but but I think with the peculiar problem of the double burden that we have, you know, both underweight and overweight and then micronutrient deficiencies, which are common in both group | I think. You know, biologically, I feel like the pre/periconceptional period. And this is based mostly on, like, you know, fully deficiency and neural tube defects risk. I think the 3 months pre and 1st 3 months of the you know, embryonic period. 1st trimester seems like a good window. But, as we were discussing previously, I don't know if a 3 month intervention will get them to a nutritional plane which it is sufficient. But is that if that period is sufficient? And so I think in the studies we do in the interventions. That we test not in a programmatic context, there's still a knowledge gap to fill and perhaps doing something even that is status linked. Or you know, it could be like, it's anaemia as the outcome, or something like that to see, like. you know, 4 months of supplementation, 3 months, 4 months, 6 months, and see if there's a change a difference, you see. So, to better define that preconceptional period. | I think we've sort of done multiple micronutrients to death | And even other micronutrients like I saw a lot of studies even done on IFA-supplements, So, iron and folic acid supplements. But I did not see a lot of studies that were conducted on calcium or vitamin D or other vitamins and minerals in general... Even iodine, iodine, I think, has been studied quite a lot, but selenium is something that I did not see a lot. Even as ascorbic acid or vitamin C, so you know, there are a lot of micronutrients that they can actually focus on and come up with a lot of interventional studies based on that |  |
|  |  | Needing to understand the aetiology of anaemia | When we say anaemia, we just think it's iron deficiency and that was the whole point in kind of understanding do all women in India who have anaemia are iron deficient. So, but what we found from our work is that only less than 50% of the women are just around 50% of the women have iron deficiency. | a collaboration for reproductive age group. We need to understand anaemia aetiologies and other micronutrient deficiencies, and I think that's the approach that we need. We need to work across South Asia, build a good collaboration | So, if the intervention says give iron to everyone, I would be very worried because of our current work where we see that women with haemoglobinopathies present with low haemoglobin but high circulating iron, and they are at risk of iron overload and then presenting with cardiac problems and heart failure. So, testing is absolutely, | we found that, you know, 27% of the mother had mild and moderate food insecurity. And there were 36% of the mother who were underweight. And mild anaemia was present in 15% and moderate anaemia in 78%. So, almost 92% of the mothers were anaemic. | Well, I suppose in South Asia the one of the major problems is anaemia. And sadly, that has hardly changed. I think over 2 decades it's still many, I don't know what percentage, but it's very high it's about 50% I think, or maybe more of adolescents are anaemic, s |  |
|  |  | Rapid nutrition transition (Over and underweight together) | We didn't focus too much on the overnutrition component and that you know that in countries like, even even in India also, that is very rapidly progressing... I know some studies are going on, but that are more mostly related to counselling, so I don't know. Some studies are going on I know. So, that is a component I think we need to focus a little bit more regarding the research perspective. | And it's a rapid, rapid transition that's happening, which is going to have major impacts upon preconception nutrition year by year. I mean we we just found in, in the population of working in Jharkhand state, in India, that the proportions of women and of small children under three who are consuming unhealthy deep fried foods and sugar sweetened foods over 2 year periods just leapt up. | so so you've got both extremes | Uh, there was a change in traditional diets to what they considered more calorie dense diets - less fresh food, more processed food |  |  |
|  |  | Routine data coverage of BMI and Hb needed | You know at the health facility. if there was some kind of little package of, I don't know, supplements of additional food or a sort of medical check up that was free of costs for every newly wedded couple to make them come. actually need to look at the details of that program in Sri Lanka, that there where they've been doing tha | low BMI has one set of problems, problems and high BMI has another, so it's sort of optimizing the BMI is what is is, what's gonna be needed. | tracking of individual women is very important. So, for example, say if you are managing, if you are treating a woman with moderate anaemia, which, say, haemoglobin level of eight or 8.5, you are giving interventions, then you have to have a mechanism to track that woman. what is her haemoglobin level after three months of therapy, whether she has improved or not, if she has not improved, what are the potential reasons try to address that... if you give iron not 100% of women will recover within three months or four months, there will be a certain proportion of 20-25% will not improve. What are the reasons? | know what gets measured gets managed or what, what's counted counts. |  |  |
|  |  |  |  |  |  |  |  |  |
|  | Challenges implementing preconception programmes | Demand side barriers (lack of awareness in population) | I don't think the barriers are that problematic. Actually, I think you just need to make people aware of it because obviously it's not something that's been on people's radar very much and.  I would if you start with contraception. And put preconception health on an equal footing with contraception. So, preventing pregnancies is is preparing for pregnancy is, as seen, seen as as important as preventing pregnancy. |  |  |  |  |  |
|  |  | Extreme weather events affecting implementation | So, we are very mountainous, difficult geography country with a very difficult and scattered settlement and sometimes we have monsoon, we have flash floods and then roads are cut off and then supply chain is disrupted. So, we do have these kind of challenges and which is one reason why people eat, you know this these non-perishable staples. | with climate change issues coming through the forefront and the effects of climate amplified. You know, there's still that seasonality, but now the effects are stronger and more intense. The rhythm is still there, but the amplitude of the exposures I think is changing and how to deal with those exposures and understand what they mean for different outcomes is really complex. | So, we are very mountainous, difficult geography country with a very difficult and scattered settlement and sometimes we have monsoon, we have flash floods and then roads are cut off and then supply chain is disrupted.  So, we do have these kind of challenges and which is one reason why people eat, you know this these non-perishable staples. | So, this summer, monsoons, having this supply chain and chain supply related challenges, this is definitely there. But then again I want to mention that this in itself does not lead to food insecurity. Sort of challenges in the country, but then like I mentioned, the nutrition security is always insecurity is always there. |  |  |
|  |  | Lack of accountability / regulation | There is no accountability in the system. Just you need to distribute. You need to distribute. Once they come. What has happened, whether the mother had received. Whether you see taking. so there is no really accountability on the part of the system. |  |  |  |  |  |
|  |  | Lack of evidence on what works despite good gov. motivation | I do not think that there's an apathy or lack of willingness among the policymakers. It's just that as researchers, we are not able to do our job. Or that is funders, a lot of old, the old policy organizations or UNICEF, or WHO? They're not really trying to do work in terms of generating evidence before starting to implement. Now I can tell you today, no, you need to give iron to everyone starting from the preconception period, and if I have a very good rapport with you or with you, would put that on your report and say ohh, this is the recommendation. But if I don't put an evidence underneath that, I don't think that's right. | the main policy issue is like kind of confusion about how to what periconceptional nutrition is and what to deliver, how to deliver and for what duration it should be delivered. So, those kind of issues need to be addressed before making it a broader policy agenda so. So, you mentioned earlier there is no uniform intervention or uniformity about interventions and what should be done. So, there is like lack of knowledge about that as well. So, that's why like this is not yet a priority at the policymaking level | there was no evidence so as because now the evidence is coming, people will think about it and definitely whatever instant it had to be implemented in the policy, I hope that subsequently it will be resolved. | yeah, I think so because because see for a policymakers, they want to know. They always wanted to know that. What is the, What is the evidence why you are saying that this is important? What is the cost for that? Because everything has cost. Again, I'm saying so two things. What? What is the evidence and what is the way that that evidence is cost effective or not? Now we are having both these evidences | So, you know, so like I mentioned in the earlier discussion, there is a huge gap of knowledge and awareness among the policymakers and the government when it comes to preconception nutrition. So, spreading the awareness about it, spreading the science behind why it is really required at that level is very important. |  |
|  |  |  | It you know, I think there's certainly been a lot of interest in recent years otherwise we wouldn't have had a program like HeLTI being funded by national funding agencies and looking at it as a 10 year program. | Mean one is what actually works and what's the timing of it? | My nutritional colleagues hate that comment, but I I still have yet to see population level evidence of dietary change and its impact on how status | Feeding programs are are showing modest effects, but honestly they're not very impressive. I mean, they're not solving the problem | we are stuck in the same guidance and the same guidelines that have been given to us since the 1970s? There hasn't been any improvement in terms of reducing anaemia or the prevalence of it, at least in India, as far as I understand. So, you can't be starting - and policies have to change and I don't think policymakers can do anything without good studies. |  |
|  |  | Lack of preconception policy | And the reason they stated for this was that there was no clear guideline which was given by the government when it comes to like when it came to preconception nutrition | there are many programs and policies that are there for pregnant and lactating women, but there is very few policies and programs which involves preconception nutrition. | the main policy issue is that there is no preconception system right now as a system level. So, that remains the prime challenge. . |  |  |  |
|  |  | Long-term programming is expensive which leads to government and donor resistance’ | And and we gotta get we gotta get donors on board here. Cause. Yeah, the donorship and resistant to thinking about this and funding the necessary studies that are that are required to get this done to generate that evidence, so we need to get them on board with this process. You know Gates and CIFF and you know all these guys have been kinda pushing back a little bit on this, you know, and I think they need to, they need to get on boar | We all live in three to five year intervals, right? When it comes to grant funding, so you can't do a long long term study with one grant. | in an ideal world to getting, you know, longitudinal trans funded. But the reality is quite different |  |  |  |
|  |  | Political structures & red tape | Second thing is there is no, there's a huge gap between the centre and the state policies, or you know the centre and the state government ruling | And in Maharashtra state, in fact, we have started it, but the pace is not as desired for various reasons. As I said, the government change. And when the government change they have, there's some agendas | he third one really is popularity, or you know that that this is, you know, electoral cycles are short..., the truth is, governments don't think a long time ahead. | But you've got something here which is, you know, a short term deliverable. Young people would will be grateful for a government that says we can make your kids healthier and we can make you healthier in pregnancy and maybe save your life. And you know, that's the sort of short term immediate issue that governments should resonate with. Umm, so you know, I think that that that strategy needs to be developed in the right sort of way and it has to come from young people. |  |  |
|  |  |  | The 1st question is, what does it cost? And it's too expensive. Immediately. Even with MMS. It became like such an important thing to make it as cheap as iron and folic acid for it to even be considered. And so the work that went on was really about like trying to get that supplement to be low, low, low cost. I think political will in general for women's nutrition - women and girls' nutrition is lacking! I just feel like it's not, It's not an affordability issue in many contexts. Like some of the governments, the countries in South Asia should be able to afford to do better with women's health and nutrition, and it's just of the lack of, you know, commitment and political will. | So, when, whenever, like you talk about the policymaker here or anywhere in the world, the first thing they are asking what will be the cost of that? | It's a very large country, so it takes time to have the desired impact in difficult areas, and I'm sure if the evidence fails and some technocrats, if they insist, if they constantly pursue government, government is at least willing to do willing to start. And in Maharashtra state, in fact, we have started it, but the pace is not as desired for various reasons. As I said, the government change. And when the government change they have, there's some agendas |  |  |  |
| How to intervene in preconception nutrition | How to reach preconception recipients | Interpregnancy period is important to meet women’s changing needs | But not just the first pregnancy, but also the repeat pregnancy. So, I think that indicates that you know in between the births they're again at that preconception period, right before the next delivery happened. So, the birth spacing I think integrating with family planning approaches and then also like those kind of areas where it's very cross cutting. | I mean, if you had enough, if you had enough time and money, I would want to look at, as you mentioned, the sort of it interpregnancy interval as well because you know, we know their pregnancy depletes woman's nutrients status and if we could supplement them through to the next pregnancy, there would be very interesting to see that impact | I think between them mainly, maybe through the healthcare system is the better because when they have the first child, then they need to have all immunization, all the health care service, then the connection with the care is more than with the.. maybe more with the healthcare and then maybe with the community, that's the two main places. | Uh, well, I think it I well, I think for women who are un, relatively undernourished, umm, that a goods, you know, 6 to 24 months of that period between pregnancies is when they're breastfeeding. So, their caloric demands and they're protein demands are high right through that entire period. Umm, so they need nutritional support anyway, right? Just because the fact that they're, you know, they're pumping out, you know, God knows how many calories every day through breast milk. Umm. And so they need nutritional support anyway. And so I think we need to think about the titration of that cause. You know, we want that the last for two years, right exclusively for six months and then continue to optimum 24 months. Optimally, umm. And and that's now approaching the desired inter pregnancy interval right ohm. So, the answer is ohh yes, of course, but we already need to supplement them. We already need to help them nutritionally because you know they're working, their bodies are working incredibly hard to generate, you know, to, you know, maintain lean body mass and to provide nutritional support for their infants. So, I would say yes, but I'm not sure what you would do other than what we should be doing already to support breastfeeding women |  |  |
|  |  | Involvement of families is important for uptake | Culturally it's not very common for the men on the husbands to get involved in the care of their wives during pregnancy or in the care of the children when they are very young. You know, that's mostly done by the mother and the grandmother, but but we actually discovered that many husbands wanted to get involved. And it was just a question of them not being able to know how to get involved so that that that's resulted in us developing 2 specific modules for husbands. So, one that focuses on their own health, but also one that focuses on how they can support their voices. | Now the woman or the family wants to wants that the that she should get pregnant. So, they may be planning pregnancy now. In that context, if you want to counsel the woman alone that no, this is not the right time because you have so many morbidities first, you should manage this morbidities or control these conditions and then you should think about the pregnancy. You can't do that alone with the only with the woman. You have to involve her husband and have to involve the family. So, for any kind of this kind of program, it is very important to involve the husband and the other family members, because sometimes in this part of the world they are the key decision makers of the family. So, it's their role is very important | No, no, the families.  Yeah, but the families, because we it's like we leave no one behind.  Kind of.  You know, if it's only between women and women, it's nothing works.  Because if the family gets that, you know, with the grasp of what nutrition means to the family, it's a good thing.  So, we would always want it.  That's the integration. | So, their parents can be addressed like or then their even their in-laws can be brought in and then we can actually ask them to send their women Umm uh, new bride to the school, so those things can be done so there cannot be like just health platform, but other platforms can also play play a part |  |  |
|  |  | Marriage as an entry point for preconception interventions (for newly weds) | I'll see because like I said, I'm most of our pregnancies are unplanned.  So, the preconception period is in ideal setting, so maybe like married woman and then like planning for pregnancy.  So, at least, yeah, three, at least three to four months. | in South Asia the rate the prevalence of early marriage is a lot.   So, I think that it's very relevant that the adolescents or Preconception, if we can get them early, that should be good enough. Because if they get married early, they're not well developed. Plus, there's some deficiency. It can be a big issue for them. | I was actually looking at some of the data we have. And so what I saw is like there are women who are getting married. One, is they're getting married early. So, like less than like about 80% of our study population, they got married before the age of 20. So, they're all in the adolescent group, right? So, that's a big chunk of women! | I think this is South Asia and sub-Saharan Africa is that, is that the 1st pregnancy is occurring among adolescents. you're beginning a reproductive cycle during that time. | And then of course, as I just talked to you that early marriage, early birth in South Asia is something we really need to consider. | 50% women are married before 16.3 years of age. So, this is like so and the problem with Bangladesh is that like 75% of women who got married, they stopped their education. So, that's why, like targeting through school based platform or education based platforms, so that is a bit problematic for our case |
|  |  |  | Like the only thing I can think of really is, is this sort of newlywed package type of thing. And maybe couple counselling of some kind.  Umm, possibly a male outreach worker? | There's a local government community health worker system where the the community health workers also maintain records of what they call eligible couples, so they have a record of newly married couples in the village and they actually maintain a record of them. And then monitor them to see if they get pregnant. There's not much pre preconceptional, nutritional counselling or anything else taking place, but they have a record of women who have been married recently, so they actually maintain something like a marriage register. And so it would be easy to identify women who are potentially eligible for preconceptional care or counselling. | But for those out of school then I think sort of adolescent groups, a newly married adolescent groups, girls and boys groups, group approaches through peer peer mobilizers might be might be a way forward. | So, if you have a reasonable vital registration system that has a marriage licensing of some kind. Umm, I would try to go after that, but you know, thinking about where we work in Sarlahi, I mean not we would just go basically house to house and identifying people who are newly married and enrol them and then sort of keep moving around the communities and then identifying newly married adding them into like an open cohort. Umm, but you know, to be fair, so my hat is more on the if efficacy side of the question rather than effectiveness. | The newlywed couples coming to you coming to the centres that they register immediately and they come for screening. I I've seen how she Lanka does it. So, after married, they just come and then they get a screening and their screen for not iron deficiency, I think they do get screening for anaemia, BMI and all of that, and I think somebody's trying to do something like that in Andhra Pradesh as well in India. |  |
|  |  |  | Umm, so I think this idea of maybe going after umm, newly married is 1 possibility. Although like how do identify who's newly married is maybe an issue you'd have to sort of be living in the community and sort of knowing or having like in Nepal with the FCHV program, those guys, they know their people, they know who's getting married and so on. But then you're sort of putting on unpaid volunteers more stuff to do. So, I you know, again, I worry about that | Well, I, I mean it's it's challenging because of these new rules around age at marriage and you know, umm, people wanting to hide that they're marrying their girls early. So, I it's I feel that there needs to be a a marriage registration system so that communities are able to identify newly weds and without the fear of retribution and sort of legal action and all of that. Because I feel like that is actually a bit of a barrier to the most vulnerable getting services. | I think it would be more challenging in, in in urban areas, but you they do still have in, taking Nepal as an example, they do still have female community health volunteers that that's working urban areas, I think.  So, they might be able to be identifying newly wedded couples in in their areas.  I do think it's more challenging but maybe if there was a package of benefits, people would go to and report from themselves to get it | I think something that engages a couple at at the newlywed period is probably the best window. | they have a record of women who have been married recently, so they actually maintain something like a marriage register. And so it would be easy to identify women who are potentially eligible for preconceptional care or counselling. |  |
|  |  | Peer networks for mobilising adolescents | For example, in the case of adolescence, if you see anything that is a school based intervention like midday meal program or you know anaemia Mukt Bharat which is a part of the National Health mission. So, these are a few programs, even Poshan Abhiyan which came in after the Modi government came in. Uh, So, even Poshan Abhiyan can be used in order to promote these things. So, this would be for the adolescent age group and also ensuring that they're doing it at the school level involving the peers, you know, having workshops along with the peer group. So, doing such kind of things is I think will help in educating them better because if you are educating the peers, you're also educating the all the other peers around them, when it comes to that age group, you know, so you can sort of motivate them and encourage encourage them to have a behavioural change when it comes to these things. So, that would be the adolescent group part of it | But for those out of school then I think sort of adolescent groups, a newly married adolescent groups, girls and boys groups, group approaches through peer peer mobilizers might be might be a way forward. | But perhaps, somebody who's in adolescent themselves might make it a bit easier for for the adolescents to interact with. |  |  |  |
|  |  | Need ‘outside the box thinking’ to engage community | maybe through the media, through the media, you know. Tell them about you know the importance of the nutrition through the television,. Or maybe by putting advertisements in the public system or public places where you know easily they are visited by many, Â like religious places. Religious places, they can also be used for reaching them. | But I think it we need to think out of the box. We cannot be stuck in the same process and the same cycle of thinking and just the same pattern, and I'm sure a lot of people will have loads of these kind of ideas which you have to I think try and probe and get out, however weird it might seem, this is where you get all the sparks. | When recall, we done an initiative where you know in the in the voice of a child.  Uh, the mothers get the uh, you know?  Call where as if the child in her room is speaking to her â€˜Mama, Have you taken your tablet today or did you read this so like that?' | name] and I talked, [name] and I talked about, you know, wouldn't it be great to get even, maybe even the fashion industry or something and you know, hire a stadium and get some celeb to come along and talk to young girls. You could possibly link it around the sort of feminine hygiene kind of thing, but and then also just talk about nutrition and why you need to, you know. | everybody would come. So, I think we need a festival of some sort, these are very weird kind of recommendations probably very and very left field you might think. But I think it we need to think out of the box. We cannot be stuck in the same process and the same cycle of thinking and just the same pattern, and I'm sure a lot of people will have loads of these kind of ideas which you have to I think try and probe and get out, however weird it might seem, this is where you get all the sparks. |  |
|  | Integration across delivery platforms is needed | Community health worker roles are vital (but over burdened) | volunteer type of people who could identify newlyweds and then, you know, there being a sort of package of some package of care, you know, as some sort of community celebration type of thing that the there's newly married girl coming into the community and then, you know, there's there's some kind of package of benefits that that young people might be able to then get. I think that might be a way, but it would need to be through a community worker I think possibly and adolescent worker, a bit like a Community health volunteer. | there's usually one lady health worker per thousand individuals within a village, and I know that's not necessarily the case in all settings in South Asia that they'd have this sort of program. | I think that men and boys are harder to reach than women because you know women, well women eventually get pregnant, of course. And then, then they're they're, they are going to be captured then. So, there's already health workers who are focused upon women. You know you've got your auxiliary nurse midwives, you've got female community health volunteers. You've got women's groups which exist in a lot of settings. And so it's, it's sort of a matter of widening the window of time that to try and find girls and women who who are younger or who are not pregnant but are intending to have a baby. | So, if that cannot be done, then at least do it at the healthcare worker level or at the Community level by providing them the preconception care at a much earlier age | And 2nd is this lady health worker program, whatever it is, but it's only the 65% actually the area population is covered by a LHW not the 100%, but at least in the beginning we can reach up to 65% and others through the health facility or this base program. |  |
|  |  |  | The adolescents out of school will be in the community. The community. Soon to be married adolescents, just married, in between pregnancy. They're all in the community. | So, that would be, I think one way to do it, because ownership when it comes to such thing, ownership plays a very important role. So, ensuring that that ownership is also there at the ground level amongst the healthcare workers who are trying to promote these things should be there. | one idea I did have was, you know, was to form a sort of an interpersonal relation, or to have that rapport with women at the ground level, at the individual level, because this is something that I observed, that if you're going to them, and if you're talking to them at a personal level and if you're trying to relate to whatever they are going through, they open up more to you. They start having more faith in, you know, they start trusting you more, you know so. So, I think that is something that even community health workers should be trained on when it comes to preconception nutrition. You know, maintaining that interpersonal rapport with them is very important. | Because, for example in India, if I thought of a preconceptional program we would probably be using the same community health workers to deliver that, but they're already quite busy. You know, they have a lot on their plate, so if we have to add preconception nutrition to them, then do we have additional, do we have additional resources to recruit more staff? Can we justify that? if we do justify that do we have the economic data to back that, or if we feel this is a priority, then what can they draw? | we still have about a third of all pregnancies taking place in girls under 18 years of age and sadly still many under 15 years of age. So, I mean, there is this ground reality where you do have to wait for a third of the cases, preconception care with the care and health of adolescents, which does mean that you have to look at platforms which are both community and educational platforms because that's where most of them are. |  |
|  |  | School platform is important but risks missing vulnerable | Yes, uh, I think definitely school-based platform is some place that you can reach them more effectively with mass amount of people that way. And then community. But if you,.. if we .. based on the recently married, it'll be little bit tricky, because, not like in the past, that they have very strict about like not being pregnant before married, nowadays they are more freedom. | the adolescent are the one, which is little difficult. But you know that that also can be reached through the same health system, because they are also staying in the community. and maybe the additional way of reaching to them is maybe through their schools and colleges where you know the special programs can be organized to reach them and then sensitize them. Or to provide them with the information regarding the importance of nutrition on their health as well as their offspring, to be born when they get married. | But as far as the boys are concerned... maybe in the school itself, when there are any functions organized. maybe those are functions can also act aa you know the opportunity to target the boys. | I mean to be easy, the school based would be the most kind of feasible one. But when we do that, we also need to think of how we can randomize. Because if we randomize within school or within class, it may have the what is that called the cross one so it it mess up.  You know what I mean? Like spill over. Contamination. Yeah, it may be contaminated if we do it. | And you know, and you have to find a place where they're not doing a midday meal program, right?  So, I mean you you want to get them to a point where you know the vast majority of these kids are coming in without any breakfast when they show up at school.  So, you gotta do something kind of early in the morning to make sure that that, that supplement actually helps them stay awake and pay attention during school and everything like tha |  |
|  |  |  | 50% women are married before 16.3 years of age. So, this is like so and the problem with Bangladesh is that like 75% of women who got married, they stopped their education. So, that's why, like targeting through school based platform or education based platforms, so that is a bit problematic for our case | And then there's there's the sort of period of adolescence when when girls may not yet be married, they may still be in school, or they may be out of school because of having been excluded from school. But they're not yet married. But that's when they're they're nutritional status is already beginning to fail, and their their suffering with their anaemia or or low BMI or other micronutrient deficiencies. So, there's that, that's a different window again and that would, I think require either school or out of school programs, which are nutritional based programs | n rural communities, 50% of our girls are out of school.  So, you need some community approaches like we here we have the lady health worker. Both the program performance is not very great, but still we have one force who can approach to these communities.  So, this is the one, but for boys, actually the schools are the best way because 70-80% boys are in the school, so you can through education system or through schools you can approach the boys easily as compared to girls.  As I told you in some places at least girls' education is is not so so high, specially in rural areas. | Well, I think you know one of the great things, you know, one of the real accomplishments over the last 20 or 30 years has been the massive increase in primary school enrolment. You know, both gross and net enrolment rates. So, I think there are I think there is a period here where you actually get to pull this off in primary school. Like the real question is what happens when they finish primary school and there are only two years into your intervention and they now go on to middle school but they don't or they drop out, right? And that's gonna require some, you know, household level ohm supplementation. | I look as well because like majority of women, their continuing, they're not continuing their education after after marriage.  So, we need to target them in the community instead of like through any any education platform |  |
|  |  | [Evidence for organising theme] | now maybe if there are better interventions, maybe it requires a some SBC social behaviour change.  So, probably we should, you know, not just not just has health system but then also as education and food system | So, we don't need to retain them in the educational settings, but like the other 75% can be targeted in the additional settings yeah and like I mean bringing the connection between the social protection program, we know you know like that in Bangladesh like if a girl goes to school, gets the stipends  And so that's also umm, I'm, I mean, everyone gets the stipend so those kind of program can be like coordinated in a way that it it improves preconceptional nutrition as an outcome as well | It it has to be tied together, we have schools and then we have the antenatal care. There has to be this link bridging everybody through schools up to being, you know till the age of 50. | do think that there is a need for a range of strategies, poverty alleviation and education being a dominant one, |  |  |
|  |  | Government influencing the private sector through taxation and regulation | So, if we know it works, you know we can think about other approaches, you know, certification, micronutrient rich foods being made freely available or something at the latest stage.  But at the moment it's still a proof of concept study, so we want to see if micronutrient supplementation actually makes a difference | And then another area is, you know the packets are there food packets, can they, you know, do they actually read the fine prints of what it is? They they they don't. So, how to you know what is the status of that? So, those are so much as printed. If there is a kind of a Mark there that you know something to do with high protein food substance for example, or high iron content and what they should be getting, this is the ideal thing for you. If there is some quality stamp on it, maybe you know that will improve because all the research initiatives have to go towards what? When what can we do to improve the status quo? Improve the current scenario. So, that's only as far as my immediate thinking of the research goes. |  |  |  |  |
|  |  |  | And of course, the taxation type of thing might be a way into ohm policy, because obviously it raises money.  So, then you can begin to talk about how the the funding that was raised by such and such programs could be deployed to train community health workers or whatever it is. | either providing the food for free that you think is useful or making that food much cheaper for, you know, certain groups of people | You, you asking governments and companies to change their behaviour, I guess, and but it, but perhaps that's more doable, particularly in certain circumstances. | requires a combination of approaches one of those, particularly in the school settings it requires look at school food environment. It requires, you know, broad legislative strategies that is for example marketing restrictions on ultra processed foods. It also requires, in my opinion, a tremendous amount of community advocacy and education in this space |  |  |
|  |  |  | tax benefits for our for some companies for getting involved in charitable missions and particularly health promotions. So, not all, but some might be persuaded to think about how some initiatives around, probably largely the health and nutrition of young girls in the workforce, might actually be something they'd like to take on | Then I suppose you need to think, how's it, how would it be solved in terms of the provision of whether it's a nutritional supplement or, umm, a change in diet, probably it's supplement.  Umm, so that might involve retailers, or it might involve primary healthcare. | Well, you know, there's always this issue of the private sector engagement, right? And local food companies and What's available in the market? | mean, you could argue this research on with the private sector on how it's provided, but you know it's such a I mean that's obviously something we're talking about here. Should the the sugar tax be extended to - WHO saying maybe it should be extended to not just the sweet drinks, but to cakes and biscuits. | So, that you're not asking people to really change their behaviour.  Makes it easier.  You, you asking governments and companies to change their behaviour, I guess, and but it, but perhaps that's more doable, particularly in certain circumstances. |  |
|  |  | Integration within the health system (particularly family planning) ensures continuity | And the other could be a family planning you know, program where women are coming to good family planning methods but when they stop wanting to be on them, that's when they could be targeted for something. If they said their intention was to become pregnant. | I think there we can definitely lean on what is being done in the family planning sector, right? Because, ideally, what do we want? We want these pregnancies to be wanted, the second ones, or even the 1st ones. And we wanted want it to be a couple's decision. And it needs to be, you know, based on., you know, being on a family being on contraception and then coming off of it, because you have planned the pregnancy right. And if you had that you would be able to know who's like. Come off their, you know, Depo-Provera, or who's not getting their con, whatever you know, oral contraception. And then at that point, in time, you could kind of say, Okay, here is an entry point. Begin, you know, counselling or talking about diets, or giving some supplements, or whatever. | I think integrating with family planning approaches and then also like those kind of areas where it's very cross cutting. | The preconceptual, the usual complaint by the clinician, is that: when do you tell them? Where do you tell them? They are not with us. You know that is their common grouse, but anybody who's coming for a contraceptive advice, anybody who is potentially in the reproductive age group, she's potentially in the preconception period. We should tell them that's what the FIGO Wellness wheel says. She may come to you for anything, but use that opportunity to guide her for this. | And then I feel another large audience, Faith, is those who go to the infertility clinics. They're going there because they cannot conceive easily. They are in the preconception bracket to every infertility specialist should be, you know, telling them a lot of things. | few adolescents, if any, go to the health system. They are generally very reluctant in terms of going to, you know, facilities with obstetricians and gynaecologists. No young girl wants to be seen outside in a gynaecologist's office, and neither do their parents want them to be there. |
|  |  |  | Of course, if you integrate into education then you may not be able to target the whole spectrum of population... whereas if you integrate that into a national health system or national health program you can Â reach all the population, the whole of the population. Right from to be married, married, pregnant between pregnancy, adolescents because they're all in the community. So, I think the best way is to integrate that into you know the existing health system | So, one is like I mean health sector can play a part in because in this South Asian country settings like the Primary Health care setting is, I mean to some extent developed and also like the women and children.  I mean, historically they are the focus of uh health sector delivery.  So, health centre can play play a part | . It is easy to put into, integrate into a national health system. Because you, you have already existing the infrastructure and the human resources. Yeah, so it would be much easier to integrate that into a national existing national health programs, or the system. | Otherwise, life course looks like life course, and you know the quick fix the problem at any point in time, even you know somebody comes with heavy menstrual bleeding.  They only fix the heavy menstrual bleeding.  It is leading to anaemia.  They're not thinking about anaemia and nutritional counselling, for example | for me it's just very simple. Two sides of the same coin: trying to help people avoid the pregnancies they don't want and have the pregnancies they do want in the best kind of health, best shape possible. | The the other is, you know whenever you are working with the public health system though the human resources in the public health system are overburdened. Provide both curative service as well as the preventive services. Plus in addition they also need to do lot of data collection and attending many meetings. So, therefore I don't know how effective they are in delivering these interventions. |
|  |  | Social protection for targeting services to vulnerable | he usual, you know, village health, sanitation day interventions, which are are there across and I'm just talking about India here just cause I've, I I've been working in India recently so, and most of the evidence has come from India actually in the in the review that we've been looking at so, those are at the top of my mind. But some, you know, some kind of an intervention with take home rations provided through a social protection mechanism | I mean, one of the things that we've seen in Nepal because they have this cash conditional cash transfer for having antenatal care and then delivering it a facility and so on, we've seen a big impact. You know, people know about the program. They all want to get paid. Umm, it's increased. No. Has it increased the quality of the care? I don't know, but it it's increased the utilization of those services | so I do think that there is a need for a range of strategies, poverty alleviation and education being a dominant one, but then also including things that improve dietary diversification through a range of strategies like for example cash transfers, linking them to poor families, fortified staples, and importantly now these new strategies that need to happen in the context of climate change and agriculture impacts |  |  |  |
|  |  | Workplace initiatives engaging people across the life course | girls' economic empowerment is an important area. And so there, there could be such platforms where there, you know, the skill building is being done. Or yeah, there could be those types of platforms that we could think about as workplace opportunities. | So, at least the proportion of men, if not all, can be reached through the workplace intervention that that search strategy that have been tried and tested in many other programs as well. So, one way of doing that is with the work place. |  |  |  |  |
|  | Range of programmatic approaches should be taken | Conflicting opinions on supplementation, dietary and fortification approaches | You know the the other issue would be if micronutrients are helpful preconceptionally and certainly we know that for folate, probably probably true for iron.  I don't know.  You know, the other approach is is the fortification approach, which sort of helps everybody in the society, but maybe it overdoses some people you know, I don't know | I know there are issues with fortification and how to do it and you know one government sort of makes it their law and then the next government comes in and takes it away and things like that.  But and you have to have sort of a product that can be fortified and that people do all consume and you know things like that, but. | So, you probably need a multi-pronged approach which I think sort of is those nutrition specific or direct and indirect elements as well. So, supplementation being something that's direct, but then food fortification would probably be something that sort of gives everybody that little bit of a bump in their intake.  So, that could be something that's really embedded more broadly and could have a benefit for preconception nutrition. | I know there are issues with fortification and how to do it and you know one government sort of makes it their law and then the next government comes in and takes it away and things like that.  But and you have to have sort of a product that can be fortified and that people do all consume and you know things like that, but. | So, you probably need a multi-pronged approach which I think sort of is those nutrition specific or direct and indirect elements as well. So, supplementation being something that's direct, but then food fortification would probably be something that sort of gives everybody that little bit of a bump in their intake.  So, that could be something that's really embedded more broadly and could have a benefit for preconception nutrition. |  |
|  |  |  | And I have no confidence that dietary interventions are going to make any difference | Yeah, I mean we, we, we also toyed with the idea of giving these lipid based supplements and uh in a calorie rich food and then we we decided not to go down that route for two reasons.  One it becomes a product.  It's not, you know, we didn't think it was food. | I don't know the answers. I don't have all the answers, but I think that's what I would expect to see in the longer term rather than just give everyone a pill. | And you can always try to translate that to food later, but I think that, you know, you wanna make a a an efficacy statement first. You know, prove that the principle actually works before you start thinking about programmatic approaches to improving nutritional status in women. | I mean, I'm honestly worried about just dietary supplementation. Even in pregnancy, I mean, we just, we don't see that big an impact. ... And so I kind of worry that maybe preconceptual supplementation won't do that much more now. | You know the the other issue would be if micronutrients are helpful preconceptionally and certainly we know that for folate, probably probably true for iron.  I don't know.  You know, the other approach is is the fortification approach, which sort of helps everybody in the society, but maybe it overdoses some people you know, I don't know |
|  |  | Education and empowerment to create demand | Education material. You know when you're trying to spread awareness or trying to educate them about preconception nutrition or any other topic for that matter. I think in such cases use or storytelling use of the local language of what they really understand, what they relate to, that should be done in order to spread awareness and education on that and maybe even a pictorial sort of a thing can be taken into consideration and they can, you know maybe do something like act or an enactment in front of everyone in order to promote that kind of education or awareness about preconception nutrition. So, this would be one of the ways that they can spread education | You know, we needed a in between catalysts.  See if we just give the hand over the checklist to an individual, she'll, you know, take the most expected answers.  They are clever enough to do that. Whereas if you put a paramedical person or a frontline healthcare provider, or somebody who's trained in this kind of administration of this checklist, for example, she is asking them some questions in a conversational manner and she's helping her pick the right thing, then you get the right data. | And in the qualitative studies, what I observed was that several women, even after giving birth to kids, they were more like a, you know, why should we take a particular supplement?  It is like a medicine. Why should I give that medicine to the child when I'm pregnant?  So, they viewed supplements to be medications instead of viewing them as supplements instead of, you know, looking at them as a benefit for their child | So, this was one you huge gap that I observed, and even educating women or, you know, community health workers is very important when it comes to preconception nutrition because a lot of them are really not aware of what benefits it can really give to the women or to the child after birth | I don't think the barriers are that problematic. Actually, I think you just need to make people aware of it because obviously it's not something that's been on people's radar very much and.  I would if you start with contraception. And put preconception health on an equal footing with contraception. So, preventing pregnancies is is preparing for pregnancy is, as seen, seen as as important as preventing pregnancy. | communities will do what communities will do. And they will do things that they perceive to be of value and for reasons that are very different from what you want them to do. And I think one big challenge there was, I think, placing everything in the basket of birth outcomes and others for young girls, you know, makes the whole thing very unattractive in the context of their own aspirations of life and what they want. I mean, the whole project was couched into a life skills building interventions. And in hindsight, I think what we did was instead of giving them life skills, we gave them reproductive health skills. So, it's much more to life than reproductive health. |
|  |  | Food security: improving availability & access | It can be challenging for sure because you don't necessarily have access to foods. So, if you're teaching someone about what they should be eating, but they can actually put it into practice that's difficult. | There are foods available that are healthy and nutritious and have micronutrients and have protein, but poverty is such a huge challenge in that setting, that actually affording them is a different situation altogether. | So, you know the market access in remote areas was very less when it came to certain foods, because of which the diversity was also very low | So, we are very mountainous, difficult geography country with a very difficult and scattered settlement and sometimes we have monsoon, we have flash floods and then roads are cut off and then supply chain is disrupted.  So, we do have these kind of challenges and which is one reason why people eat, you know this these non-perishable staples. | And then poverty alleviation strategies, just cause accessing healthy and nutritious foods is a challenge. So, how can you help families with that? |  |
|  |  |  |  | The first is that basically our experience is that, the women are poor and their main concern is nutrition and the women are poor there for sometimes they are unable to afford. And this is reflected in what do we call this in all these states, we have below poverty line. The families which are below poverty line it. We find it difficult to advise them and also we find difficult about the compliance. It is not in the domain of health, but it is overall sector social welfare sector. There are some schemes to provide food to these women, but anyhow the uptake, the compliance is suboptimal. | So, from an individual perspective, it is very hard in our setting to access affordable, nutrient dense and healthy diets. That was a big challenge. There are foods available that are healthy and nutritious and have micronutrients and have protein, but poverty is such a huge challenge in that setting, that actually affording them is a different situation altogether. And there's been huge inflation in the past. I mean, since the pandemic inflation has been really, really big and that's made an already bad situation quite a bit worse. So, that's a really big challenge |  |  |  |
|  |  | Hesitancy around cash-based transfers | But it I unfortunately I think you'd probably have to tie it with a voucher or something to get to get people to spend it on actually, on nutritious foods for the family and for to actually consume micronutrient rich and protein-rich foods rather than, you know, just spending it on day-to-day white polished rice or that kind of thing. | there's a lot of social normative pressure. And so the study was designed to actually target newlyweds, providing them with a conditional cash incentive, and with counselling and support for family planning methods to delay that 1st pregnancy and some kind of a paraded way, such that you know. The longer they waited the more cash they would accrue. | But on the other hand, you know, maybe this goes back to your sort of social protection idea again as well.  I mean, one of the things that we've seen in Nepal because they have this cash conditional cash transfer for having antenatal care and then delivering it a facility and so on, we've seen a big impact.  You know, people know about the program.  They all want to get paid.  Umm, it's increased.  No.  Has it increased the quality of the care?  I don't know, but it it's increased the utilization of those services | Will cash incentives to households make a difference? I mean, what? You know what's the what's? What's the other options that can improve that they can improve the nutritional status of adolescents, but you know, till we have some evidence that in a controlled setting it really works. All that stuff has the potential to kind of just get washed away and you see no impact, right? Did you not see any impact? You have no idea what the story is, right? You don't know why there wasn't an impac | I think it puts more income into a household and you would hope that that would sort of translate into improved diets of the girls and perhaps boys preconceptionally umm. But it's sort of, it seems like a more diffuse pathway. |  |
|  |  | SBC to address complex drivers of preconception malnutrition | the adolescent period has probably 2 important aspects for the health of their own selves rather than thinking about future generations.  We have done some work with adolescents and I think for behaviour change interventions particularly adolescence might be a good time to start those interventions because if, if, if, if some of those can be established at that age, then I think there's a chance it will actually carry on through.  And if at some point in the future they decide to have children, then that's absolutely fine | So, that was one very surprising thing, and dietary diversity was not seen in the entire region.  I just saw them having only rice and finger Millet.  Largely there were no other cereals like even wheat was not consumed very regularly in that area.  So, you know, so there was very less dietary diversity, not just with seeds and nuts of fruits and vegetables, but also with cereals.  Although India has so many cereals, you know, like so many millets, which they can have on a regular basis, but there was literally just maybe 1% of the entire sample that I took had good diversity when it came to cereals | I think the cultural belief and practice is also something we need to consider because a lot of regions is some, uh, some belief that some food are hot, some food are cold.  What [to] consume what not [to] consume, particularly when they get pregnant? | Umm, now a lot of our population is converting to vegetarianism, so I think there is also a lot of drive and because of all the social media and all this and people have access to all sort of information, some incorrect.  So, now, especially in the females on there is a sort of a a behavioural change and the diet pattern is changing to vegetarianism, and this is a very huge challenge for Bhutan. | And I have no confidence that dietary interventions are going to make any difference | And #2 be and we have always been trying to do this, but then maybe we are making a very gradual and unnoticeable progress, but then trying to diversify our diets.  This is one major challenge - Inclusion of more fruits and vegetables, animal source proteins, dairy. So, this is a very big challenge in the country. |
|  |  |  | I mean, you'd have to improve the at least in South Asia, you would have to improve the status of young women in the household. | OK, so nobody allowed the plantation of the trees. For example here so so many of these misconceptions which are there which also needs to be, you know, taken into account for a robust implementation. So, the gap is not so much in our understanding of what nutrition is all about. To put the policies may be easier than implementing it as is the case with many of the things with implementation, so the more barriers will stand in the way of the nutrition related because it is so behaviour based. | Plus, if they add the daughter in law the right, there's some kind of accessibility and the what is that - the disparity and and like household? Because sometimes the the daughter you know cannot eat all the good one but the mother in law, all the husband need to eat 1st and then eat after that. | I have seen that women or people in general find it very difficult to stick on to eating healthier or doing physical activities on a regular basis. So, that behaviour change and the reinforcement of that behaviour till when should the reinforcement be done? | What factors drive diet and physical activity patterns of adolescence and a what interventions can we potentially?  Develop and we realize that it's quite important to do it with them rather than just give them an intervention or in an administered and intervention.  But but I think behaviour change interventions, particularly with |  |
|  |  | Social media promoting preconception | And in addition, again, same thing with the giving ads in the televisions or advertisement in the television and displaying the you know the matter in the public places. I think that should work well. | So, it is now the demand generation in that space is happening more and more. And also because the digital space and social media and then women are not producing dozens of babies, just one or two. So, they want to be careful even ahead of pregnancy. | We have to build the capacity for that and use the electronic space or digital space very cleverly to position some of these awareness initiatives and to make them realize that you must ask this question to your doctor. | everybody is using this social media platform in the country. Almost everybody uses this, especially the youth and adolescent population. They are really into social media platforms and in keeping with times the Department of Public Health, we also have all our social media handles in almost all the social media platforms trying to reach out to this population and Umm, the department is also in the process of developing an app for the youth to cater to youth and adolescent to cater to their their specific needs. And then through this app there will be getting all the required information for them on Â sexual reproductive health including you know, nutrition and preconception nutrition | There are the two ways. One you know about this is the maybe the social media that is the electronic media and all the social media.  You know is is very powerful. Every household at least have the mobile phone and so the TV is available in every householder |  |
|  |  |  | spreading the right kind of messages through social media campaigns or through WhatsApp, because WhatsApp is something that everyone uses. So, maybe spreading spreading messages through that can also help is what I feel when it comes to preconception nutritio | More of you know the tiktok type of ads and the social media.  Just 10 seconds, which holds their interest, but 1 point,1 point gets driven or a gamification in in an engaging mann |  |  |  |  |
|  |  | Strengthening provision of preconception services from health workers | The clinician doesn't have the time for that, so there is a whole spectrum of, you know, things that we can do both on the supply side. But for the, you know, the quality of what kind of advice is given, we have to build the capacity for that and use the electronic space or digital space very cleverly to position some of these awareness initiatives and to make them realize that you must ask this question to your doctor. You know they must ask something, though they generally ask, and we generally say something. That's not it at all. So, the supply side should be strengthened. And the demand is I feel it's easier to generate because people do want to take care every day | And that includes reference to the FIGO nutrition checklist, which we set up and which we pioneered in a whole range of countries now and and that's including in India... you need to engage with the local healthcare community and say 'look, we want you to help us with this, don't worry, we're not giving you too much extra work, we're giving you the resources, this doesn't take long to do. Women and their partners actually can do it themselves to a degree, and you know we're giving you guidance and advice and extra information for any problems that you pick up'. | And unfortunately, because of the kind of way that they're trained, when they talk to young couples about it, it's very much a one way communication of transmission. | So, educating them about this is extremely important and in order to increase the knowledge of preconception nutrition among them, one very good way would be mainly maybe to, you know, come up with some sort of an education material from the government's end and making sure that healthcare workers are also educated and made aware of preconception nutrition | Then the person needs to be able to say something. Then the care provider needs to be confident and equipped to say something sensible |  |
|  |  | Use of apps to improve health behaviour preconceptually | And then they can always, in this era of the digital space, they can always say, OK, go and seek out from this app, you'll get your recipes, you'll get your know how of how many calories, what you're eating. The person, you know has the opportunity to go in depth, though... the demand is I feel it's easier to generate because people do want to take care every day |  |  |  |  |  |
|  |  |  |  |  |  |  |  |  |
|  | Different research approaches identified | Collaborative network is key to coordinate research | We have identified the people we've identified champions like Vani says, and the people are really willing to do this work. I think we just this whole kind of network that we have built just needs to kind of come in contact with somebody who is able to then give that funding for this network to do the work. It has to be a South Asia wide collaboration is not one individual, not MaatHRI, not you know ICDI, not Aga Khan, it has to be a South Asia collaborative and somebody will and UNICEF is best place to bring everybody together. | Maybe you should should have some expert group meetings where you can and and in that meeting it's not only the people who are in academics or in Research even who are in the program, they should come and share their thoughts about the potential research gaps... And from that, I think 1 White paper or a concept note of the future potential research questions to be addressed in this domain should come because this because there are lots of other issues | So, I think there is a momentum and I can only kind of hope and be very, very optimistic that things will change and it will improve and things will only turn out to be better and hopefully we'll be out of this status quo and the cycle of things that we are stuck stuck with at the moment. | Then for this South Asian countries like developing like different intervention packages with some similarity of course but like different intervention packages, considering the context in this countries and also our regional differences within the country is also important |  |  |
|  |  | Implementation research is needed to translate research into practice | how do you make it programmatically feasible and which are kind of see as a its own piece in a way. | And for the other two components, I'm just talking about preconception because our main discussion point, this PRECONCEPTION now. You can't go and and even a state can't implement the full package of wings in a public health program without having doing an implementation research, because there are many components. | how we translate these capacity building into actually the patients getting the benefit out of it, where is the gap? That is something that we have to do. | what we want to do is quite simple, but actually delivering it is not. And so, you know you you need to |  |  |
|  |  |  | The research I think now is implementation research.  I think we know what needs to be done.  The question is how can we do it and what works? | It's not practical. It's not pragmatic. It's not feasible. It just doesn't get off the ground. Then what is the use of, you know, making such a wonderful elaborative for this, which cannot be implemented | Just doing research if you can't scale it up and then what is the use to do that | in a scaled up system, how on Earth would something of that intensity be possible so? |  |  |
|  |  | Importance of co-designing interventions | So, in a way, we've actually co-developed the intervention and the intervention package with the community.  So, for example, during a formative work, we did a lot of focus group discussions and we did it with different groups, with the women themselves, with the mothers and their mothers in law, the husbands, the village elders, the government officials, the community health workers, et cetera, et cetera.  So, there were things that actually came up during those discussions, for example, exposure to pesticide was a big factor for them | The the second thing about adolescence, I think is it's it's very important to work with them to actually get them to co-develop the interventions with us.  And that's something again we have discovered through some of the formative work.  You know, actually did a study on adolescent | So, a part of it becomes a bit didactic, but it's mostly by working with them and helping the women arrive at their own solutions.  And what they think will work in their setting and in their specific circumstances. |  |  |  |
|  |  | Need for complex interventions | We also realized that single interventions probably only had modest effects, and focusing on Nutrition alone on micronutrient supplementation alone might not be the answer.  Because we were seeing women who had multiple challenges in a so focusing on just Nutrition alone probably wasn't the solution.  At least that's what we felt, and so therefore we decided to have a multifaceted intervention which focused on Nutrition, maternal, mental health, uh pollution, hygiene, and parenting skills and child development, all underpinned by behaviour change | In terms of the research questions, see that the point let me talk about this point that we delivered a package.  And that package worked now.  Which component of the package worked?  That is very interesting to understand. | So, you know, maybe it's a package of interventions that one has to test that are not just diet.  Umm, but sort of everything going into the pregnancy is sort of healthy. | in designing a complex intervention, you need to kind of come up with a theory of change of how a how women end up having these preconceptual nutrition problems in the 1st place, and then would be what are the various pathways by which you might influence them | So, I that's sort of the, that's one thing that I like about these interventions that have that are a package. because I do feel like they try to address multiple issues at once. Â the problem with it is if you see an impact, you don't know which components are the ones that matter most. |  |
|  |  | Need to determine cost effectiveness of interventions | You know, they have a lot on their plate, so if we have to add preconception nutrition to them, then do we have additional, do we have additional resources to recruit more staff? Can we justify that? if we do justify that do we have the economic data to back that, or if we feel this is a priority, then what can they draw? | you know, these programs cost a lot of money. unless they are well designed, well targeted, well evaluated based on robust good evidence I think it'll be a waste of money, time, energy and we will be going around in circles and in 10 years time we'll again be ohh. We are still stuck at this. These are our statistics. | he second thing is you've gotta do the economics. You've got to be able to say, and this is a lot of work we've been doing in PMCH, we've got some advocacy documents around that. That not only umm is, is there a need for greater funding and the cost benefit ratio is very high? Usually it's about 10, so for every dollar you invest, you get the return of about 10, which is a phenomenal bargain. And there are very few other things in life that give you that sort of return on investment. [talking about the UK] | I mean, you know that everything has its cost, so it's the, it's the, when whenever you talk about them that you know ministers or other parliamentarians and and other policymakers, so they are always talking about how much cost.  But there is no hurdle or resistance from the from policymakers and from other stakeholders.  everyone is actually knowing about this issue and they agreed upon this that we need some something for people before marriage out, during pregnancy or before pregnancy |  |  |
|  |  |  | we need totalk about targets. we need to look at when to start and what to start. So, that so that we optimize the cost effectiveness. Because ultimately, when it is a program the cost is a major important factor. Yes, and many times programs fail because of their high cost. | What is the cost for that?  Because everything has cost.  Again, I'm saying so two things.  What?  What is the evidence and what is the way that that evidence is cost effective or not? | The 1st question is, what does it cost? And it's too expensive. Immediately. Even with MMS. It became like such an important thing to make it as cheap as iron and folic acid for it to even be considered. And so the work that went on was really about like trying to get that supplement to be low, low, low cost. I think political will in general for women's nutrition - women and girls' nutrition is lacking! I just feel like it's not, It's not an affordability issue in many contexts. Like some of the governments, the countries in South Asia should be able to afford to do better with women's health and nutrition, and it's just of the lack of, you know, commitment and political will. | So, when, whenever, like you talk about the policymaker here or anywhere in the world, the first thing they are asking what will be the cost of that? |  |  |
|  |  | Randomised trials are needed to test interventions | So, I think it would be a, it would be good to do a trial where we actually test whether or not it has an impact. at the moment as far as I understand it, they're just doing operational research in that regard, the evidence from the studies that I have done have been only on birth outcomes in association with pregnancy exposure. | But it's very likely to have to be cluster randomized. Just be for acceptability purposes. And you might, you know that might. There might be a package there where you know certain pieces, non nutrition intervention, pieces of the package can be applied. Both the control group and to the intervention group and that might be things like you know, you know, enhanced educational things or some you know something else that's got nothing to do with nutrition. But actually that's very unlikely to improve the growth of kids, right | maybe the with some control quasi experimental rather than doing the randomized controlled trial you need a lot of resources. But if you have the resources, then maybe the RCT is the best option here. | If micronutrient with the pill, with the blinding, you know kind of thing, it may be OK, but behaviour change it's very difficult for in the same setting the right, |  |  |
